# N-terminal β-strand underpins biochemical specialization of an ATG8 isoform

**DOI:** 10.1371/journal.pbio.3000373

**Published:** 2019-07-22

**Authors:** Erin K. Zess, Cassandra Jensen, Neftaly Cruz-Mireles, Juan Carlos De la Concepcion, Jan Sklenar, Madlen Stephani, Richard Imre, Elisabeth Roitinger, Richard Hughes, Khaoula Belhaj, Karl Mechtler, Frank L. H. Menke, Tolga Bozkurt, Mark J. Banfield, Sophien Kamoun, Abbas Maqbool, Yasin F. Dagdas

**Affiliations:** 1 The Sainsbury Laboratory, University of East Anglia, Norwich, United Kingdom; 2 Department of Biological Chemistry, John Innes Centre, Norwich, United Kingdom; 3 Gregor Mendel Institute (GMI), Austrian Academy of Sciences, Vienna BioCenter (VBC), Vienna, Austria; 4 Institute of Molecular Pathology (IMP), Vienna BioCenter (VBC), Vienna, Austria; 5 Institute of Molecular Biotechnology, Austrian Academy of Sciences, Vienna BioCenter (VBC), Vienna, Austria; 6 Imperial College London, Department of Life Sciences, London, United Kingdom; Institute of Basic Medical Sciences, NORWAY

## Abstract

Autophagy-related protein 8 (ATG8) is a highly conserved ubiquitin-like protein that modulates autophagy pathways by binding autophagic membranes and a number of proteins, including cargo receptors and core autophagy components. Throughout plant evolution, ATG8 has expanded from a single protein in algae to multiple isoforms in higher plants. However, the degree to which ATG8 isoforms have functionally specialized to bind distinct proteins remains unclear. Here, we describe a comprehensive protein–protein interaction resource, obtained using in planta immunoprecipitation (IP) followed by mass spectrometry (MS), to define the potato ATG8 interactome. We discovered that ATG8 isoforms bind distinct sets of plant proteins with varying degrees of overlap. This prompted us to define the biochemical basis of ATG8 specialization by comparing two potato ATG8 isoforms using both in vivo protein interaction assays and in vitro quantitative binding affinity analyses. These experiments revealed that the N-terminal β-strand—and, in particular, a single amino acid polymorphism—underpins binding specificity to the substrate PexRD54 by shaping the hydrophobic pocket that accommodates this protein’s ATG8-interacting motif (AIM). Additional proteomics experiments indicated that the N-terminal β-strand shapes the broader ATG8 interactor profiles, defining interaction specificity with about 80 plant proteins. Our findings are consistent with the view that ATG8 isoforms comprise a layer of specificity in the regulation of selective autophagy pathways in plants.

## Abstract 2

ATG8 is a ubiquitin-like protein involved in autophagy pathways. Biochemical characterization of the expanded ATG8 gene family in plants revealed an additional layer of specificity in selective autophagy, determined by ATG8’s N-terminal β-strand.

## Introduction

Macroautophagy (hereafter autophagy) is a conserved cellular quality control pathway that removes unwanted self and non-self macromolecules to maintain homeostasis in response to physiological and environmental fluctuations [1,2]. There is a growing appreciation that autophagy is a highly selective process, with multiple layers of specificity defining the dynamics of uptake, subcellular trafficking, and turnover of autophagic substrates [3,4]. However, despite these advances, the molecular details of how various autophagy cargoes and components are recognized, recruited, and recycled remain to be fully elucidated [5,6]. In particular, our understanding of the molecular codes that define selective autophagy in plants is limited [7,8].

The autophagy machinery consists of around 40 autophagy-related proteins (ATGs), which are highly conserved across eukaryotes [2,9]. Core ATG proteins mediate the repeated insertion of the ubiquitin-like protein autophagy-related protein 8 (ATG8) into the growing autophagosome membrane, culminating in the formation of a double-membraned vesicle. Generally, these mature autophagosomes are trafficked through the cell to fuse with the vacuole, where autophagic cargoes are degraded and their building blocks—nucleic acids, amino acids, lipids, and carbohydrates—are recycled and returned to the cytoplasm [10]. Autophagy can also participate in other processes besides the recycling of cellular components. For example, recent studies have implicated autophagy in unconventional secretion of cytosolic proteins [11,12], and susceptibility to invading pathogens [13,14].

ATG8 is a key player in the selective autophagy pathway. Besides being the major structural component of autophagosome membranes, ATG8 binds selective autophagy receptors and adaptors, as well as core ATG proteins, and autophagy-specific soluble N-Ethylmaleimide-sensitive factor (NSF) attachment protein receptors (SNAREs) that mediate fusion of the autophagosome with the vacuole [10,15–17]. ATG8-interacting proteins often carry a conserved motif named the ATG8-interacting motif (AIM; also known as the LC3-interacting region [LIR] motif), which follows a W/Y/F-X-X-L/I/V consensus sequence and is typically surrounded by negatively charged residues [18]. ATG8 is composed of four α-helices and four β-strands, with a variable N-terminal extension that mediates the growth of the nascent autophagosome via fusion of ATG8-containing vesicles [19–21]. There are multiple ATG8 isoforms in metazoans that appear to be functionally specialized, based on interactome analyses [22,23] and a few recent functional studies [24–30]. One emerging view is that ATG8 specialization could form a layer of specificity in selective autophagy pathways in addition to the autophagy cargo receptors. This could occur through ATG8 interaction with different sets of proteins [22,23,31], posttranslational modifications, such as ubiquitination [26] and acetylation [32], and localization to different subcellular compartments [29,33]. However, despite these recent and consequential advances, ATG8 specificity has yet to be functionally studied in plants.

Phylogenetic analyses revealed that ATG8 has dramatically expanded throughout the evolution of land plants as compared with algae, which only carries a single ATG8 [34]. ATG8 isoforms in land plants range from 4 in rice to 22 in oilseed rape [34]. Plant ATG8s group into two major clades: Clade I contains all of the algal and most angiosperm ATG8s, whereas Clade II is unique to land plants. Interestingly, ATG8s cluster in phylogenetic groups that are specific to particular plant families, indicating that ATG8s have diversified across plant taxa. For example, Solanaceae, the family that contains potato and *Nicotiana* spp., have four well-supported monophyletic ATG8 clades that do not include ATG8s from other plant species, such as the model plant *Arabidopsis* [34]. A plausible hypothesis is that distinct ATG8 lineages have acquired specific polymorphisms throughout evolution that may have contributed to the diversification of selective autophagy pathways in plants [34]. Nonetheless, the degree to which these polymorphisms underlie ATG8 functional specialization remains unknown.

We recently showed that PexRD54, a secreted virulence protein from the Irish potato famine pathogen *Phytophthora infestans*, binds the potato ATG8 isoform ATG8-2.2 (formerly ATG8CL, Clade I) via a canonical C-terminal AIM to modulate selective autophagy in host plant cells [35,36]. We solved the crystal structure of ATG8-2.2 in complex with a 5–amino acid peptide matching the C-terminal AIM of PexRD54, and showed that this AIM peptide docks within two hydrophobic pockets, W and L, similar to the yeast and metazoan ATG8-AIM complexes [36]. Interestingly, PexRD54 has a 10-fold higher binding affinity towards ATG8-2.2 compared with the potato Clade II ATG8-4 isoform (formerly ATG8IL) [35]. Additional in planta biochemical and cell biology assays further confirmed that PexRD54 has higher affinity to ATG8-2.2 compared with ATG8-4. For instance, PexRD54 increased accumulation of ATG8-2.2 autophagosomes and rerouted them towards the pathogen interface, but failed to significantly label and perturb ATG8-4 autophagosomes [35,37]. These findings indicate that these two potato ATG8 isoforms have different biochemical activities, and prompted us to further explore the hypothesis that plant ATG8 proteins are functionally specialized.

In this study, we used quantitative biochemistry and proteomics to explore the molecular basis of ATG8 specialization in solanaceous plants. First, we used immunoprecipitation (IP) followed by mass spectrometry (MS) analyses to determine that the six ATG8 potato isoforms bind distinct sets of plant proteins, with varying degrees of overlap. Domain swaps and structure-guided mutagenesis experiments revealed that a recently derived polymorphism within the N-terminal β-strand of ATG8-4 underpins weak binding affinity towards PexRD54 and contributes to enhanced selectivity towards AIM-containing plant proteins. Our findings are a first step towards decoding the molecular signatures that define selective autophagy in plants. We propose that ATG8 specialization is an important specificity layer that determines the recruitment of cellular proteins to modulate selective autophagy pathways. In addition, the potato ATG8 interactome we produced reveals a number of novel proteins that are likely to play important roles in selective autophagy responses, thus serving as a valuable community resource and a foundation for future studies.

## Results

### Solanaceous ATG8 isoforms associate with distinct sets of proteins

**Fig 1:**
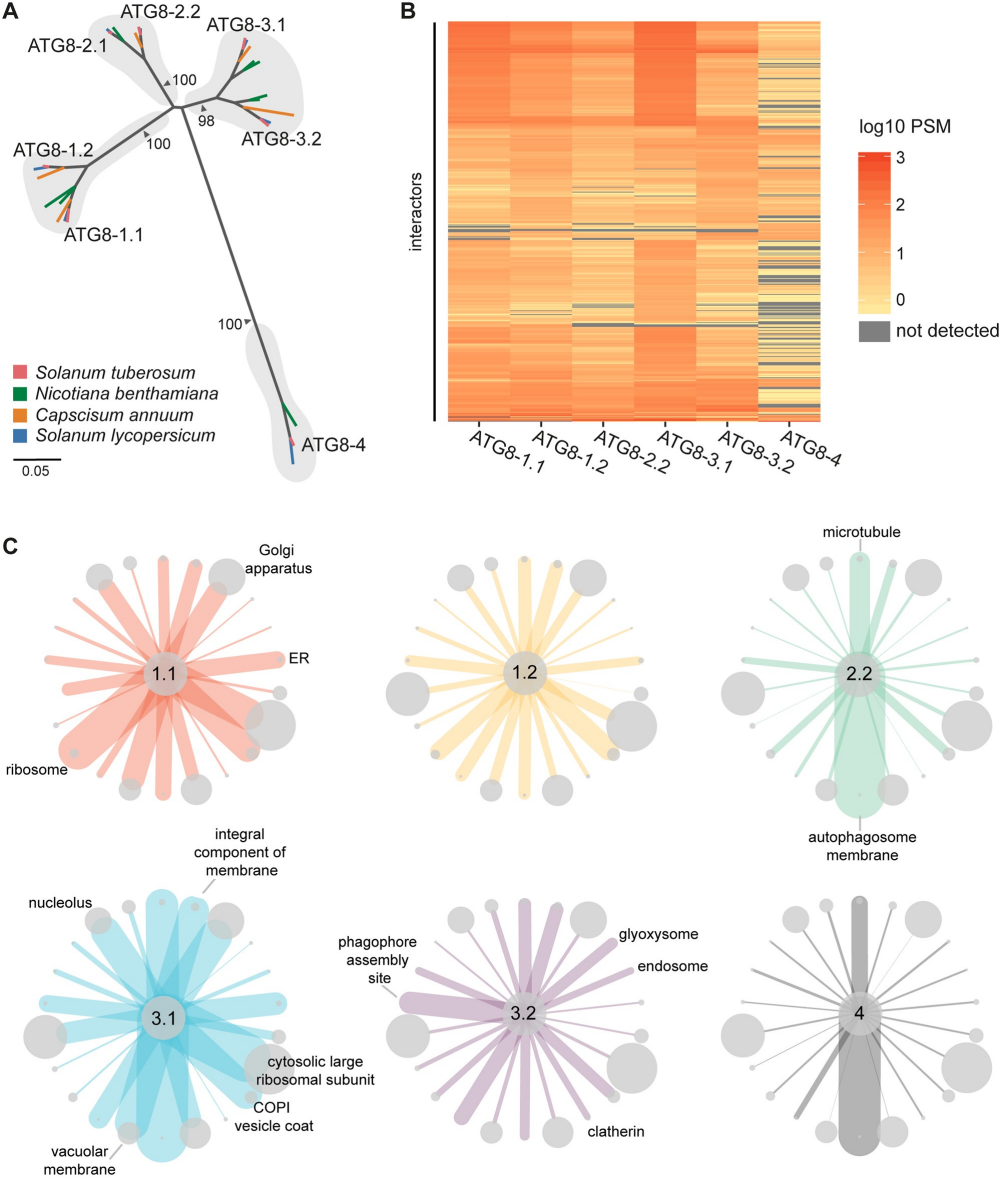
Solanaceous ATG8 isoforms have distinct plant protein interaction profiles. (a) Orthologous relationships between solanaceous ATG8 isoforms. Unrooted maximum-likelihood phylogenetic tree of 29 ATG8 isoforms, with gray shading highlighting clades, and colors indicating plant species. The tree was calculated in MEGA7 [38] from a 369-nucleotide alignment (MUSCLE [39], codon-based). The bootstrap supports of the major nodes are indicated. The scale bar indicates the evolutionary distance based on nucleotide substitution rate. (b) Heatmap showing the interaction profiles of all ATG8 isoforms. The average PSM data from two replicates were log10 normalized and then used to construct a hierarchically clustered heatmap with the scale as shown. (c) Network representation of the interactions between ATG8s and protein groups defined by GO cellular compartment annotations. Proteins were grouped based on the cellular compartment annotations, and a subset of groups were chosen for representation. The sizes of the nodes are scaled to the number of interactors in each respective group, and the edges are weighted to the average PSM values for all the interactors in each respective group for each ATG8. Nodes are labelled where the average PSM value is most differential compared with the other ATG8s. S6 Fig provides a graphical figure legend. ATG8, autophagy-related protein 8; ER, endoplasmic reticulum; GO, gene ontology; PSM, peptide-to-spectrum match.

Previous phylogenetic analyses revealed that the ATG8 family is expanded in plants and that ATG8 isoforms form well-supported family-specific clades, such as in the Solanaceae, in which ATG8s cluster into four major clades [34]. Potato encodes six ATG8 isoforms that cluster in these clades among close homologs from other solanaceous species (Fig 1a; S1 and S2 Figs). Based on sequence divergence (S3 Fig), we hypothesized that clade-specific ATG8s would interact with specific plant proteins and thus exhibit a degree of functional specialization.

To investigate this hypothesis, we determined the interactor profiles of all six potato ATG8s using IP coupled with MS following transient expression in *Nicotiana benthamiana*. Our interactome analyses included two replicates and revealed 621 proteins that associated with at least one ATG8 isoform but not with the empty vector control (S1 Table; S4 Fig). Consistent with our hypothesis, the potato ATG8s associated with unique complements of proteins with varying degrees of similarity, with ATG8-4 exhibiting the most selectivity (Fig 1b; S5 Fig). The ATG8s showed differential interactions with a number of protein sets defined by cellular compartments (Fig 1c, S6 Fig) and biological processes (S7 Fig), with the remaining sets common to all ATG8s. Within the interactome, we detected several core autophagy proteins and known ATG8-interacting proteins, including endogenous ATG8s, validating our IP-MS approach (S1 Table; S8 Fig). Moreover, about half (48%) of proteins in our dataset had closely related proteins within the human ATG8 interactome defined by Behrends and colleagues (2010) [22] using IP-MS—a figure that is markedly higher compared with random sets of proteins of the same size (621 proteins) (S9 Fig; S2 Table).

We also captured a number of vesicle trafficking-related proteins, including Rab GTPases, Rab GTPase-activating proteins, endosomal sorting complex required for transport (ESCRT) complex subunits, myosins, clathrin, and coatomer subunits (S1 Table) that were also identified in interactome studies using human ATG8 isoforms [22,23]. Excitingly, we found several hitherto unknown ATG8-associated proteins, including proteins that are predicted to localize to various organelles, making this dataset a useful community resource for future functional studies interrogating organelle recycling (S1 Table). Around 40% of all interactors are predicted to contain AIMs, of which a majority (73%) are conserved in either *Arabidopsis thaliana* or *Marchantia polymorpha*, suggesting the reliability of this dataset to further selective autophagy studies in plants (S1 and S3 Tables).

Overall, the IP-MS screen indicated that solanaceous ATG8s have distinct interactor profiles, with ATG8-4 showing the most striking pattern. This prompted us to hypothesize that certain ATG8 domains or residues determine substrate binding specificity and underpin ATG8 specialization.

### ATG8 isoforms show differential binding to PexRD54

**Fig 2:**
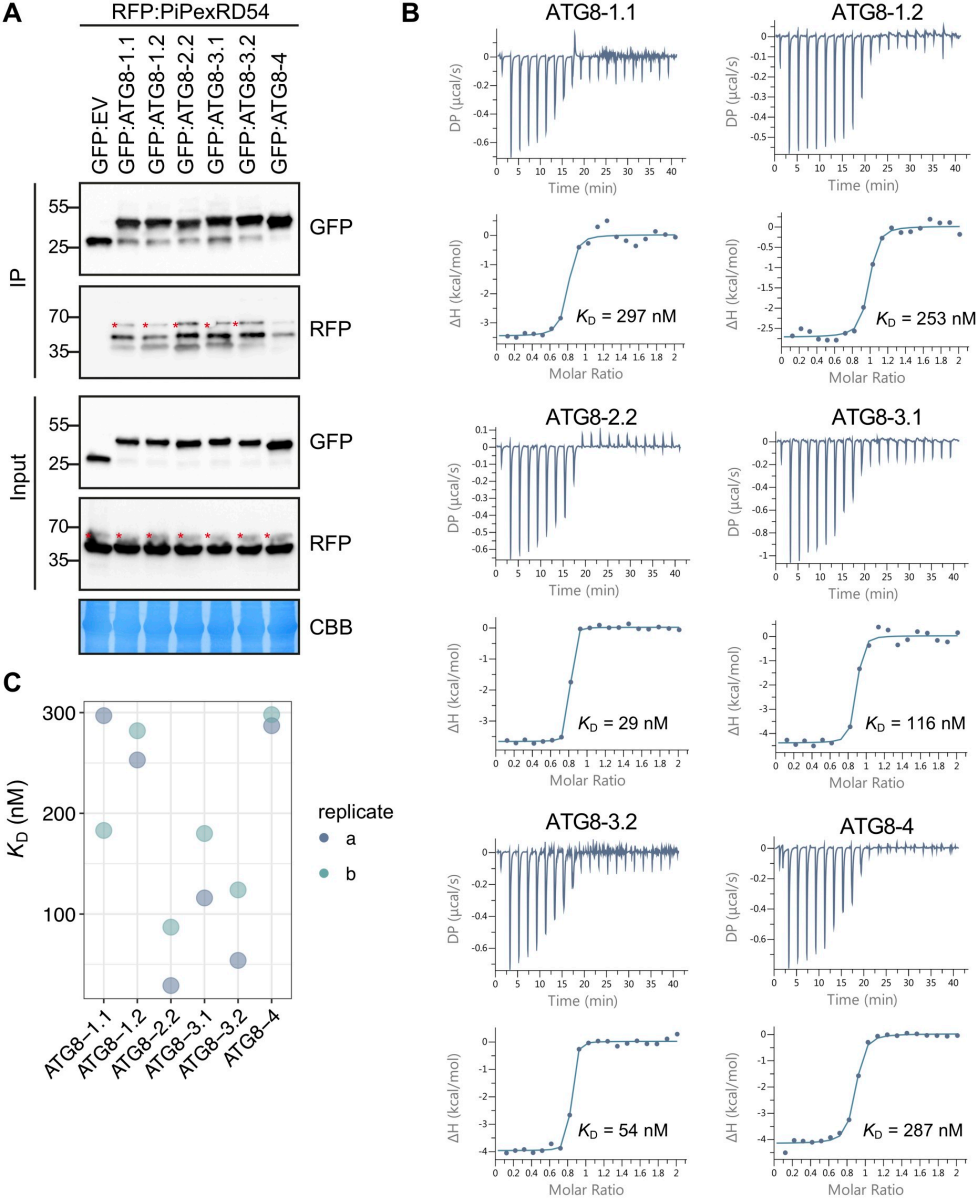
ATG8 isoforms show differential binding to PexRD54. (a) Co-IP experiment between PexRD54 and all potato ATG8 isoforms. RFP:PiPexRD54 was transiently co-expressed with GFP:EV and all potato GFP:ATG8s. IPs were obtained with anti-GFP antiserum, and total protein extracts were immunoblotted with appropriate antisera (listed on the right). Stars indicate expected band sizes. (b) The binding affinities between ATG8 isoforms and the PexRD54 AIM peptide were determined using ITC. The top panels show heat differences upon injection of peptide ligands and lower panels show integrated heats of injection (•) and the best fit (solid line) to a single site binding model using MicroCal PEAQ-ITC analysis software. (c) Chart summarizing the *K*_D_ value for each interaction across two replicates and highlighting variation within the replicates. AIM, ATG8-interacting motif; ATG8, autophagy-related protein 8; CBB, Coomassie brilliant blue; Co-IP, co-immunoprecipitation; GFP:EV, green fluorescent protein empty vector; IP, immunoprecipitate; ITC, isothermal titration calorimetry; PiPexRD54, *P*. *infestans* PexRD54.

Our previous studies have shown that ATG8-2.2 and ATG8-4 have clear differences in binding affinity towards PexRD54. Therefore, we decided to use PexRD54 as a tool to dissect the structural elements within potato ATG8s that determine binding specificity to AIM-containing substrates. The potato ATG8s showed a range of association strengths with PexRD54 in in planta co-immunoprecipitation (Co-IP) experiments, indicating the capacity for ATG8s to selectively bind the same substrate (Fig 2a). To quantify these affinity differences in vitro, we expressed and purified all potato ATG8 isoforms from *Escherichia coli* (S10 Fig). We then tested these ATG8 isoforms for binding with the PexRD54 AIM peptide using isothermal titration calorimetry (ITC) experiments. The ATG8 isoforms interacted with the AIM peptide with varying degrees of strength ranging from 29 nM to 287 nM (Fig 2b; S10 Fig). We then performed an additional biological replicate of our ITC experiments, in which we obtained similar binding affinities (Fig 2c; S10 Fig). Overall, ITC affinity measurements correlated with the trends in binding strength observed in the Co-IP experiments, in which we used the full-length PexRD54 protein, indicating that the AIM peptide alone is sufficient to recapitulate ATG8 binding specificities. In both methods, ATG8-2.2 and ATG8-4 displayed the greatest difference in PexRD54 binding strength, in a range that is consistent with our previous findings [35,36] (Fig 2c).

### The first β-strand of ATG8 underpins discriminatory binding to PexRD54

**Fig 3:**
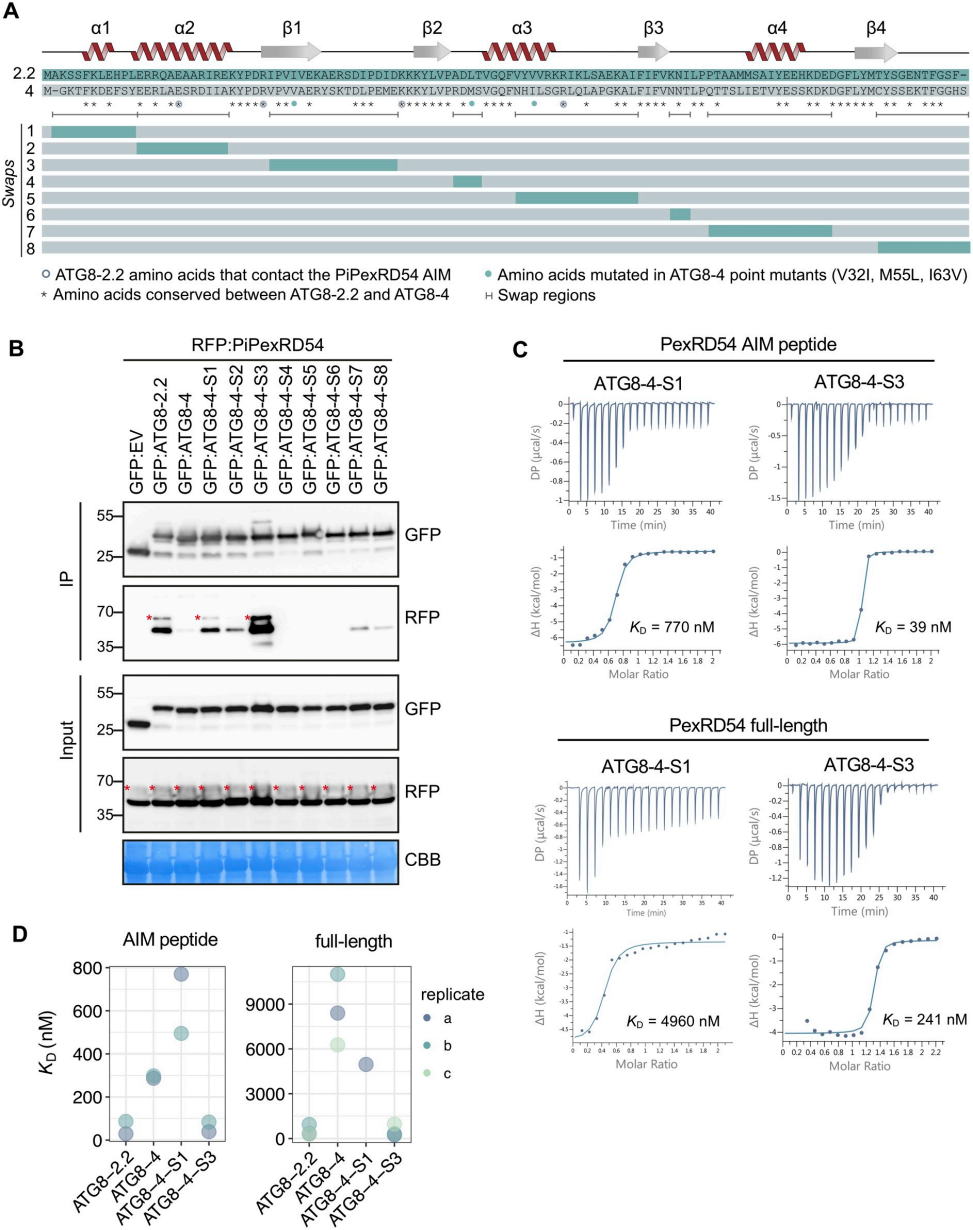
The ATG8 region surrounding the first β-strand is responsible for discriminatory binding to PexRD54. (a) Schematic showing the ATG8 swap chimeras and point mutants. The amino acid sequences of ATG8-2.2 and ATG8-4 are aligned, with the protein model above corresponding to the ATG8-2.2 structure. The brackets beneath the alignment indicate the boundaries of the swaps, with the color-coded rectangles below showing the chimeras made for each swap. The symbols beneath the alignment correspond to different features of the sequences: (i) the asterisks (*) mark conserved residues between ATG8-2.2 and ATG8-4, (ii) open circles mark residues that directly contact the PiPexRD54 AIM, and (iii) the filled circles mark the ATG8-4 residues used in structure guided mutagenesis experiments to match ATG8-2.2 (V32I, M55L, I63V). (b) Co-IP experiment between PexRD54 and all ATG8 swap chimeras. RFP:PiPexRD54 was transiently co-expressed with the controls GFP:EV, GFP:ATG8-2.2, and GFP:ATG8-4, and all of the GFP:ATG8 swap chimeras (Swaps 1–8). Immunoprecipitates (IPs) were obtained with anti-GFP antibody and total protein extracts were immunoblotted with appropriate antibodies (listed on the right). Stars indicate expected band sizes. (c) The binding affinities of ATG8-4-S1 and ATG8-4-S3 towards PexRD54 AIM peptide and full-length PexRD54 were determined using ITC. The top panels show heat differences upon interaction and lower panels show integrated heats of injection (•) and the best fit (solid line) to a single site binding model using MicroCal PEAQ-ITC analysis software. (d) Chart summarizing the *K*_D_ values for all ATG8 swap chimera interactions tested, including two replicates with the PexRD54 AIM peptide and three replicates with full-length PexRD54. AIM, ATG8-interacting motif; ATG8, autophagy-related protein 8; CBB, Coomassie brilliant blue; Co-IP, co-immunoprecipitation; GFP:EV, green fluorescent protein empty vector; IP, immunoprecipitate; ITC, isothermal titration calorimetry; I63V, isoleucine 63 to valine; M55L, methionine 55 to leucine; PiPexRD54, *P*. *infestans* PexRD54; V32I, valine 32 to isoleucine.

To further investigate the ATG8 structural features that underpin discriminatory binding to PexRD54, and, by proxy, AIM-containing substrates, we generated chimeric proteins between ATG8-2.2 and ATG8-4 (Fig 3a). We sequentially replaced ATG8-4 domains, the weakest PexRD54 interactor, with the corresponding domains from ATG8-2.2, the strongest interactor. Altogether, we obtained a suite of eight ATG8 chimeras (Swaps 1–8) and assayed these for gain of binding to PexRD54 (Fig 3a). In Co-IP experiments, ATG8 chimera swap 3 (ATG8-4-S3), encompassing the first β-strand (β1), restored binding to PexRD54 (Fig 3b). ATG8 chimera swap 1 (ATG8-4-S1) showed weak interaction with PexRD54, although this result was not consistent across replicates (Fig 3b).

To validate these results using quantitative assays, we purified ATG8-4-S1 and ATG8-4-S3 from *E*. *coli* and tested for binding to both PexRD54 full-length proteins and the AIM peptide (S11 Fig). Consistent with the Co-IP results, ITC measurements showed that the chimera ATG8-4-S3 bound to PexRD54 full-length protein as well as the AIM peptide with a similar affinity as ATG8-2.2, whereas ATG8-4-S1 bound weakly (Fig 3c). We repeated these experiments and obtained similar results (Fig 3d; S11 Fig). Together, these results indicate that the ATG8 region encompassing β1 is crucial for binding to PexRD54, specifically via this substrate’s AIM.

### A single residue in β1 underpins discriminatory binding to PexRD54

**Fig 4:**
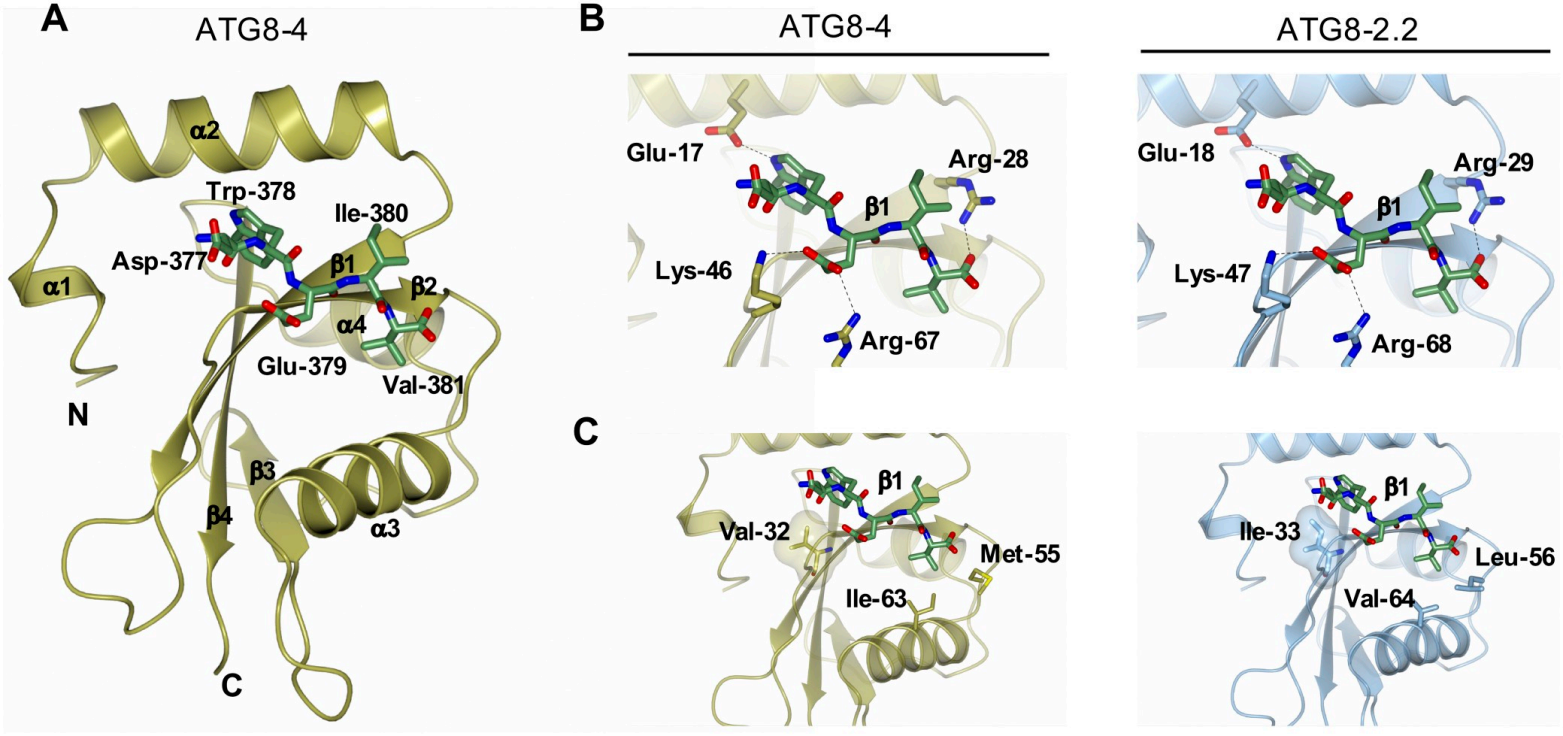
A Comparison of ATG8-2.2 structure and ATG8-4 model identifies polymorphic residues within the AIM binding site. (A) Homology model of ATG8-4 and PexRD54 AIM peptide complex. ATG8-4 and PexRD54 AIM are illustrated in cartoon and stick representation. α-helices, β-strands, and N and C termini of ATG8-4 are labelled. (B) Zoomed-in view of the AIM peptide binding pockets of ATG8-4 (left) and ATG8-2.2 (right), with amino acids making electrostatic interactions (dashed lines) labelled. (c) Zoomed-in view of the AIM binding pocket of ATG8-4 (left) and ATG8-2.2 (right), highlighting differential residues contributing to hydrophobic interactions with the PexRD54 AIM peptide. AIM, ATG8-interacting motif.

In parallel, to compare the AIM binding pockets of ATG8-2.2 and ATG8-4, we generated a homology model for the ATG8-4–AIM peptide complex using our previous ATG8-2.2–AIM peptide complex as a template. ATG8-4 adopts a globular structure composed of a C-terminal ubiquitin-like domain and an N-terminal helical domain consisting of tandem α helices (α1 and α2) (Fig 4a). Close inspection of the ATG8-4–AIM peptide complex revealed that the AIM peptide is anchored in a cavity at the surface of ATG8-4 via (i) electrostatic interactions with ATG8-4 residues and (ii) burial of AIM hydrophobic residues in two pockets of ATG8-4 (Fig 4b and 4c). The Trp-378 of the AIM peptide sits in a hydrophobic pocket located between α2 and β1 of ATG8-4, whereas Val-381 resides in a distinct hydrophobic pocket between β2 and α3 (Fig 4a and 4b; S12 Fig).

Comparison of the AIM binding pockets of ATG8-2.2 and ATG8-4 revealed that amino acids mediating electrostatic interactions are conserved between both ATG8s (Fig 4b). However, three residues that contribute to the hydrophobic pocket that accommodates the AIM peptide are polymorphic between ATG8-2.2 and ATG8-4 (Fig 4c). Isoleucine 33 (Ile-33), which is located in the W pocket of ATG8-2.2, is changed to Val-32 in ATG8-4. Similarly, Leu-56 and Val-64, located in the Val-381 binding pocket of ATG8-2.2, are replaced by Met-55 and Ile-63, respectively. In sum, our structural analysis suggested that three polymorphic residues between ATG8-4 and ATG8-2.2 could contribute to the differential interactions with PexRD54 AIM.

**Fig 5:**
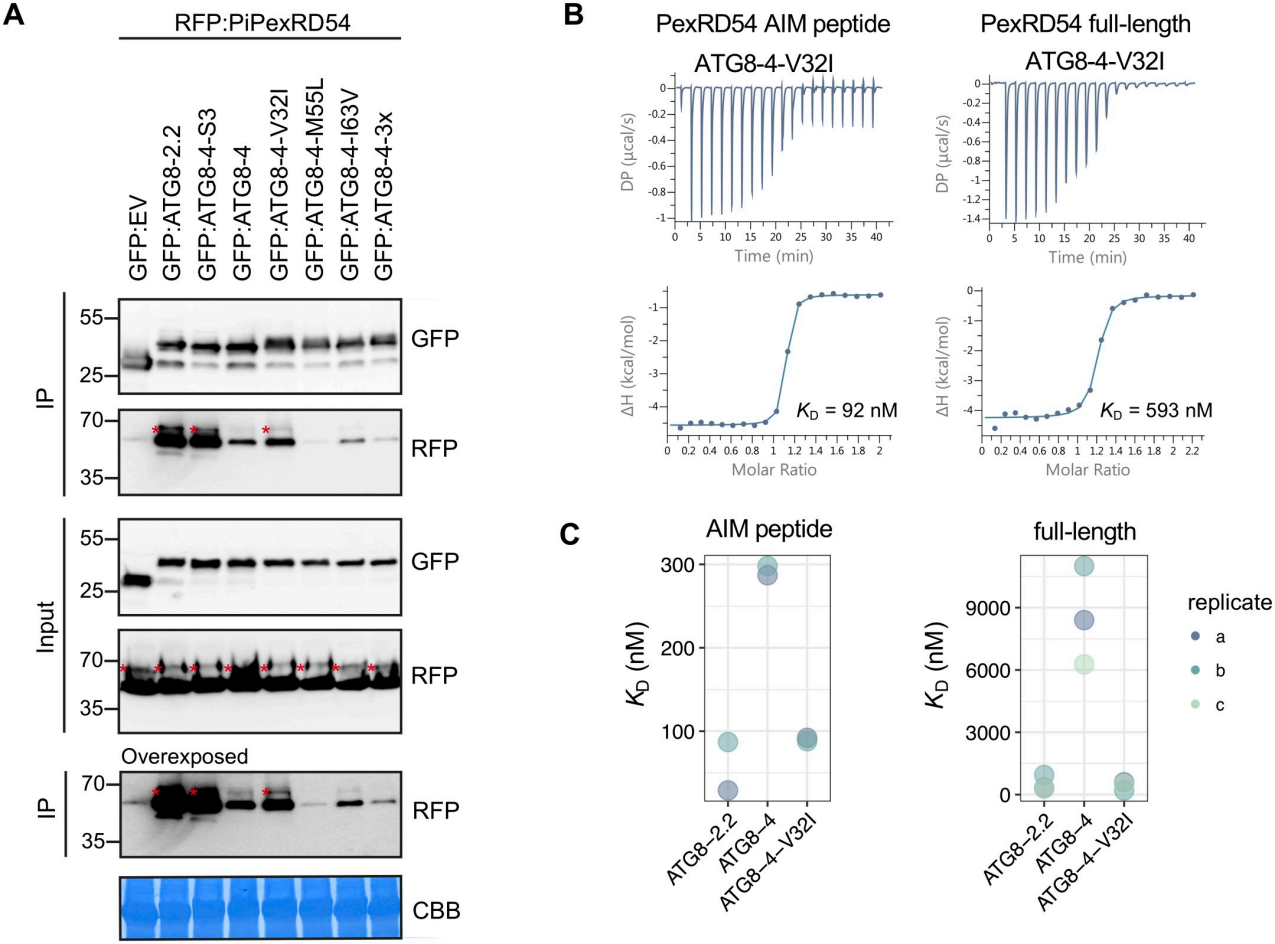
A single amino acid residue, Val-32 in the first β-strand, determines differential binding affinity of ATG8-4 towards PexRD54. (A) Co-IP experiment between PexRD54 and all ATG8-4 point mutants. RFP:PiPexRD54 was transiently co-expressed with the controls GFP:EV, GFP:ATG8-2.2, GFP:ATG8-4-S3, and GFP:ATG8-4 and all of the GFP:ATG8-4 point mutants. IPs were obtained with anti-GFP antibody, and total protein extracts were immunoblotted with appropriate antisera (listed on the right of each). Stars indicate expected band sizes. (B) The binding affinity of ATG8-4-V32I with PexRD54-AIM peptide and full-length PexRD54 was determined by ITC. The top panels show heat differences upon injection of ligands and lower panels show integrated heats of injection (•) and the best fit (solid line) to a single site binding model using MicroCal PEAQ-ITC analysis software. (C) Chart summarizing the *K*_D_ values for each interaction, including two replicates with the PexRD54 AIM peptide and three replicates with full-length PexRD54. ATG8, autophagy-related protein 8; CBB, Coomassie brilliant blue; Co-IP, co-immunoprecipitation; GFP:EV, green fluorescent protein empty vector; IP, immunoprecipitate; ITC, isothermal titration calorimetry; PiPexRD54, *P*. *infestans* PexRD54.

To test if these residues underpin the binding specificity, we mutated each of these residues in the ATG8-4 background to match ATG8-2.2 (Fig 3a). We also generated a combined triple mutant (ATG8-4-3x) and assayed all of these variants for gain of binding to PexRD54 (Fig 5a). The ATG8-4 point mutant Val-32 to Ile-32 (ATG8-4-V32I), within the first β-strand, partially restored binding to PexRD54 in Co-IP experiments (Fig 5a). We then purified the ATG8-4-V32I variant and quantified the gain-of-binding phenotype using ITC (S13 Fig). Remarkably, with both the PexRD54 AIM peptide and the full-length PexRD54, the ATG8-4-V32I mutant showed a strong gain-of-binding phenotype, restoring binding to levels that are similar to ATG8-2.2 (Fig 5b and 5c). We obtained similar results in multiple biological replicates (S13 Fig). Altogether, these results suggest that a single amino acid residue, Val-32 in the first β-strand, largely determines the differential binding affinity of ATG8-4 towards PexRD54.

### The N-terminal β-strand defines the protein interactor profiles of ATG8-2.2 and ATG8-4

**Fig 6:**
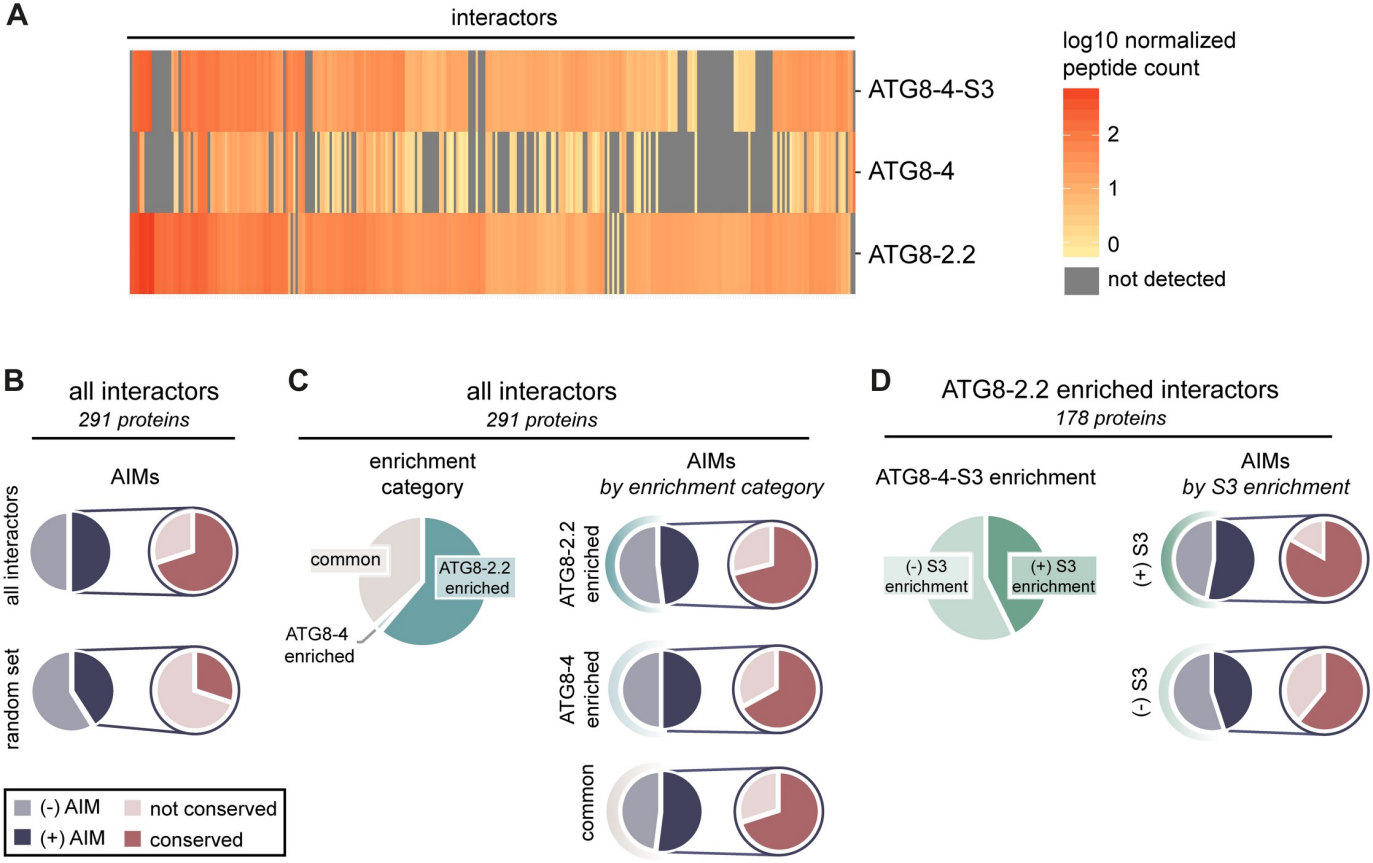
The first β-strand defines the AIM-dependent interaction profiles of ATG8 isoforms. (A) Heatmap showing the interaction profiles of ATG8-2.2, ATG8-4, and ATG8-4-S3. The average peptide count data from three replicates was log10 normalized and then used to construct a hierarchically clustered heatmap with the scale as shown. (B) All interactors in the dataset (291 proteins) and their closest *A*. *thaliana* and *M*. *polymorpha* homologs were analyzed for predicted AIMs using iLIR software [40]. The proportion of interactors that contain a predicted AIM—as well as the proportion of those AIMs that are conserved—is summarized. These are compared with the analogous values calculated from the average of three sets of random proteins from the *N*. *benthamiana* proteome (291 proteins/set). (C) All interactors were divided into enrichment categories based on whether they showed a significantly (*p* < 0.05) stronger interaction with ATG8-2.2 or ATG8-4, as determined by an ANOVA with a post hoc Tukey test; interactors that showed no significant difference in their interaction with either protein were categorized as “common.” For each enrichment category, the proportion of interactors that contain a predicted AIM, and those AIMs that are conserved, are summarized. (D) For each interactor enriched in ATG8-2.2 pull-downs, we determined whether ATG8-4-S3 showed a significant (*p* < 0.05) difference in its interaction strength compared with ATG8-2.2 using an ANOVA with a post hoc Tukey test. Proteins that showed no statistical difference in their interaction with ATG8-4-S3 compared with ATG8-2.2 are categorized as “(+) S3 enrichment”; those that showed a statistical difference are categorized as “(−) S3 enrichment.” The proportion of interactors that contain a predicted AIM, and those AIMs that are conserved, are summarized for each ATG8-4-S3 enrichment category. AIM, ATG8-interacting motif; ATG8, autophagy-related protein 8.

Because we found that the first β-strand contributes to selective binding to the AIM-containing substrate PexRD54, we hypothesized that this region also underpins binding specificity to other ATG8-interacting proteins. To test this, we performed in planta IP with tandem MS (IP-MS) experiments with ATG8-4-S3, ATG8-2.2, and ATG8-4 in three biological replicates, resulting in a final list of 291 proteins. First, we detected significant overlap with our ATG8 interactome data (approximately 40% of interactors), validating our IP-MS approach (S4 Table). We then interrogated this dataset to see if we could identify interactors enriched for interaction with either of the ATG8 isoforms. Similar to our first ATG8 interactome screen, ATG8-2.2 and ATG8-4 associated with distinct sets of proteins (Fig 6a). Close to two thirds of the proteins in the dataset were found to be significantly enriched in their interaction with either ATG8-2.2 (177 proteins) or ATG8-4 (6 proteins) using an ANOVA analysis with a post hoc Tukey honestly significant difference (HSD), while remaining proteins similarly interacted with both isoforms (105 proteins) (S5 Table; Fig 6c). Much like the ATG8 interactome dataset, this dataset had an overrepresentation of predicted AIM-containing proteins (50%), with a majority of those AIMs evolutionarily conserved (70%), as compared with a random set of *N*. *benthamiana* proteins of the same size (35% and 27%, respectively) (Fig 6b; S5 and S6 Tables).

We then compared the interaction profile of ATG8-4-S3 with those of ATG8-2.2 and ATG8-4. The ATG8-4-S3 interaction profile more closely resembled ATG8-2.2 than ATG8-4, indicating that inclusion of the first β-strand from ATG8-2.2 in the ATG8-4 background was sufficient to shift the specificity of the resulting chimera (Fig 6a). ATG8-4-S3 associated with around 40% of the proteins that were significantly enriched in the ATG8-2.2 pull-down to a level statistically indistinguishable from ATG8-2.2 (Fig 6d; S14 Fig). Within this set of ATG8-2.2– and ATG8-4-S3–enriched proteins, there was an overrepresentation of predicted AIM-containing proteins (53%), of which a majority of AIMs were conserved (83%), compared with the ATG8-2.2 enriched proteins that ATG8-4-S3 does not interact with (45% and 61%, respectively) (Fig 6d). In addition, ATG8-4-S3 does not interact with two of the six ATG8-4–enriched interactors, and maintains similar interaction with all interactors common to both ATG8-2.2 and ATG8-4 (S14 Fig). To see if the interactors significantly enriched in both the ATG8-2.2 and ATG8-4-S3 pull-downs had any specific properties, we performed gene ontology (GO) analyses, homology searches, and localization predictions (S5 Table). These analyses revealed that there are no other obvious trends within the group of proteins that seem to show a preference for interaction with ATG8-4-S3, besides the overrepresentation of proteins with evolutionarily conserved AIMs (S5 Table). In addition, the role of the N-terminal β-strand region in conferring binding to AIM-containing proteins was confirmed for one of the interactors identified in the IP-MS screen, vacuolar protein sorting 4 (Vps4), providing further validation for the dataset (S15 Fig).

## Discussion

Over the last decade, our understanding of autophagy has evolved from a starvation-induced bulk degradation process to a highly selective cellular homeostasis pathway [6]. This prompts the question—what are the molecular codes that define selective autophagy pathways? To date, the majority of the selective autophagy studies ascribed specificity to the cargo receptors that bind ATG8 via the conserved AIM [3]. In this study, we provide evidence that biochemical specialization of ATG8s is another layer of specificity that may contribute to functional specialization and subcellular compartmentalization of autophagy. This view is consistent with the evolutionary history of plant ATG8s, which have dramatically expanded in land plants and exhibit family-specific clades that differ in fixed amino acid polymorphisms [34]. We also build on this view by producing an interactome resource for potato ATG8s that serves as a platform for further studies on the diversification of selective autophagy pathways.

As a part of our study, we generated a comprehensive ATG8 interactome resource for the six potato ATG8s. We validated the quality of our ATG8 interactome data in different ways. First, the results between the biological replicates were positively correlated, confirming reproducibility of our results (S4 Fig). We also noted that the ATG8 interactome contains most of the known ATG8-interacting proteins, such as core autophagy proteins ATG4 and ATG7, and autophagy receptor NBR1 (S1 Table). Moreover, four of the endogenous *N*. *benthamiana* ATG8s were present in the interactome (S1 Table). One of the ATG8s, *N*. *benthamiana* ATG8-4 (NbATG8-4), exhibited a selective interaction pattern, interacting almost exclusively with ATG8-4, its closest homolog (S8 Fig), supporting our model that autophagosomes likely carry different populations of ATG8s. The interactome also has several vesicle trafficking components, such as Rab GTPase-activating proteins and coatomer subunits, which were also uncovered in human ATG8 interactome studies [22,23]. Moreover, the large amount of related proteins shared between this ATG8 interactome and the human ATG8 interactome described by Behrends and colleagues (2010) further points to the reliability of this dataset and may also reflect the consistency in the types of proteins detected by IP-MS (S2 Table, S9 Fig). The dataset also contains highly abundant proteins such as ribosome and proteasome subunits, which are known to undergo autophagic degradation [42,43]. However, further studies will be necessary to distinguish these particular interactors from the CRAPome, the common false positive proteins observed in IP experiments [44].

Interestingly, we detected several proteins that have not yet been associated with autophagy. Notable interactors include dual specificity protein tyrosine kinase, as well as both web family and lipin family proteins (S1 Table). As these proteins contain conserved predicted AIMs, our interactome could serve as a great resource for future studies that aim to discover novel autophagy receptors, adaptors, and regulators. Relatedly, the overrepresentation in the dataset of interactors that contain predicted evolutionarily conserved AIMs suggests that the interactome is enriched in putative autophagic components and indicates that evolutionary conservation may be an additional parameter to predict bona fide AIM sequences. Interactors without predicted AIMs may still directly interact with ATG8s via noncanonical AIMs or other ATG8-binding motifs [45]. The ATG8 interactome described herein reflects plant autophagic cargo under specific conditions, agrobacterium infiltration and ATG8 overexpression, which likely affect the observed ATG8 proteomic profiles. Future work describing plant ATG8 interactomes under alternate conditions—such as basal cell maintenance, as well as a range of biotic and abiotic stress responses—would add to our understanding of how different ATG8 isoforms contribute to the dynamics of the selective autophagy pathway.

Recent studies indicate that human ATG8s are functionally specialized. The interactor profiles of human ATG8 isoforms has been explored using IP-MS [22,41] and proximity labeling [28] proteomics. These studies revealed limited overlap between different ATG8 isoforms, particularly with proximity labeling proteomics, which primarily identifies cargoes within various ATG8 autophagosomes [28]. In addition, mechanistic studies on ATG8-interacting proteins also support specialization of human ATG8 isoforms. For example, the xenophagy receptor NDP52 specifically binds the ATG8 protein LC3C [46], whereas both the autophagy adaptor PLEKHM1 and the tumor necrosis factor Fn14 prefer to bind a different ATG8, GABARAP [16,29]. A recent study even revealed the GABARAP interaction motif (GIM) that mediates high-affinity binding of GABARAP-interacting proteins [25]. Moreover, an additional study has shown that residues within the core AIM (in mammals, LIR) and the adjacent C-terminal region are critical for the binding of receptors and adapters to the GABARAPs [30]. Considering that some plant species have over 20 ATG8 isoforms, compared to 6 in humans, it is reasonable to hypothesize that ATG8s directly contribute to the functional diversification of selective autophagy pathways in plants. Indeed, our finding that ATG8s differentially interact with different types of cargo is consistent with this view (Fig 1c; S7 Fig).

Previous studies investigating ATG8–AIM peptide complexes have shown that hydrophobic and electrostatic interactions underlie substrate specificity of ATG8 isoforms [24,31,47]. For example, a recent study that structurally characterized the *Caenorhabditis elegans* ATG8 isoforms LGG1 and LGG2 showed that the hydrophobic AIM binding pocket and the surrounding regions determine substrate specificity [24]. In contrast, because all the residues that form electrostatic interactions are conserved between ATG82.2 and ATG8-4, our data suggest hydrophobic interactions drive the marked difference in interaction strength between ATG8-2.2 and ATG8-4 with the substrate PexRD54. Future studies will reveal whether electrostatic interactions also contribute to substrate specificity of other plant ATG8 isoforms.

Detailed analysis of the ATG8-2.2 hydrophobic pocket revealed that Ile-33—which is only a methyl group larger than the valine residue found in the ATG8-4 isoform—primarily underlies binding to PexRD54, especially in in vitro *a*ssays. The isoleucine in ATG8-2.2 may better fill the hydrophobic cavity and thus lead to stronger interactions with the PexRD54 AIM peptide. Ile-33 is highly conserved in various ATG8 isoforms [34], suggesting that the valine polymorphism (Val-32) in ATG8-4 is a recently derived polymorphism. It is tempting to speculate that this polymorphism was selected to evade PexRD54 binding and thus subversion of autophagy by the pathogen. Complementation of higher-order ATG8 mutants with valine-substituted ATG8 isoforms could challenge this hypothesis and reveal whether valine substitution has an adverse effect on autophagy in general. Interestingly, a similar isoleucine to valine polymorphism underlies the pyrabactin selectivity of the abscisic acid receptors PYL1 and PYL2 [48], highlighting how subtle differences in substrate binding pockets could lead to functional diversity.

The ATG8 N-terminal β-strand also underpins binding specificity to plant substrates, with polymorphisms in this region accounting for differential interaction with about 80 plant proteins. Proteins significantly enriched in both the ATG8-2.2 and ATG8-4-S3 pull-downs, compared with ATG8-4, had a higher proportion of predicted conserved AIMs (44%) than proteins that were enriched in ATG8-2.2 but not ATG8-4-S3 (27%). This suggests the N-terminal β-strand mediates discriminatory binding to AIM-containing substrates, a conclusion further supported by the validation experiments carried out with Vps4, an interactor identified in the IP-MS screen (S15 Fig).

Vps4—also known as suppressor of K^+^ transport growth defect 1 (SKD1) in *A*. *thaliana*—is essential for the function of the ESCRT and is required for multivesicular body formation [49]. Although previous research has linked ESCRT components to autophagic degradation in plants [50], the molecular basis of this connection was not clear. Our results reveal a direct interaction between Vps4 and ATG8. Considered together with the recent studies in metazoans revealing a role for ESCRT complex in phagophore closure [51,52], our results provide a basis for future studies to further investigate the interplay between the ESCRT complex and autophagy pathways. Our findings also demonstrate how to leverage the IP-MS data presented herein to identify novel autophagic cargoes or regulators.

In summary, plant ATG8 isoforms are specialized and bind distinct sets of proteins, and the hydrophobic pocket that accommodates the AIM peptide contributes to binding specificities. This suggests that multiple ATG8 isoforms should be assessed when measuring autophagy dynamics in plants. Based on this work and other studies from mammalian systems, we propose a model in which, together with the autophagy receptors, different ATG8 isoforms could contribute to the subcellular compartmentalization of various selective autophagy pathways, especially when they are active at the same time in a cell.

## Materials and methods

### Gene cloning

#### Cloning for recombinant protein production for in vitro studies

PexRD54 was cloned in a previous study [35]. DNA encoding all different members of ATG8 family from *Solanum tuberosum*, amino acid residues Ser5-Asn114 (except for ATG8-4, Thr4-Lys113) were amplified by PCR from cDNA. For gain-of-function swaps in the ATG8-4 background, amino acid residues Thr4-Lys113 (except for ATG8-4-Swap1, Ser5-Lys113) were amplified as described above. All PCR amplicons were subsequently cloned into the pOPINE vector [53] using In-Fusion cloning based on in vitro homologous recombination, using a commercial kit (Clontech, Mountain View, CA) generating uncleavable C-terminal 6×His-tagged proteins for purification. The ATG8-4-V32I mutant was custom synthesized into the pUC57-Amp vector (Genewiz, UK) and subsequently amplified and cloned into the pOPINE vector as described above. All constructs in pOPINE vectors, pOPINE-ATG8-2.2, pOPINE-ATG8-1.1, pOPINE-ATG8-1.2, pOPINE-ATG8-2.1, pOPINE-ATG8-3.1, pOPINE-ATG8-3.2, pOPINE-ATG8-4, pOPINE-ATG8-2.2, pOPINE-ATG8-4-Swap1, pOPINE-ATG8-4-Swap2, pOPINE-ATG8-4-Swap3, pOPINE-ATG8-4-Swap7, and ATG8-4-V32I, were transformed into *E*. *coli* strain BL21 (DE3) for recombinant protein production. All primers used in PCR for cloning are shown in S7 Table. All the constructs were verified by DNA sequencing.

#### Cloning for in planta Co-IPs

The ATG8 isoforms were amplified from *S*. *tuberosum* cDNA and cloned into pK7WG2 vectors as described previously for ATG82.2 [35]. ATG8-2.2 and ATG8-4 were also cloned as level 0 modules for Golden Gate cloning [54]. ATG8 swaps and ATG8-4 point mutants were synthesized as level 0 modules for Golden Gate cloning [54]. GFP:ATG8-2.2, GFP:ATG8-4, GFP:ATG8 swaps, and GFP:ATG8-4 point mutants were generated by Golden Gate assembly with pICSL13001 (long 35s promoter, The Sainsbury Laboratory [TSL] SynBio), pICSL30006 (GFP, TSL SynBio), and pICH41414 (35s terminator, TSL SynBio) into the binary vector pICH47732 [54]. All constructs were verified by DNA sequencing. The potato Vps4 (PGSC0003DMT400019113) coding sequence and the corresponding AIM mutant, Vps4^AIM^, were synthesized as entry modules for Green gate cloning [55]. Vps4:3XMyc and Vps4^AIM^:3XMyc were generated by Green gate assembly protocols, as described previously [55].

#### Transient protein expression in *N*. *benthamiana* and total protein isolation

Transient gene expression in planta was performed by delivering T-DNA constructs with *Agrobacterium tumefaciens* GV3101 strain into 3–4-week-old *N*. *benthamiana* plants as described previously [56]. *A*. *tumefaciens* strains carrying the plant expression constructs were diluted in agroinfiltration medium (10 mM MgCl_2_, 5 mM 2-(N-morpholine)-ethanesulfonic acid [MES], pH 5.6) to a final OD_600_ of 0.2, unless stated otherwise. For transient co-expression assays, *A*. *tumefaciens* strains were mixed in a 1:1 ratio. *N*. *benthamiana* leaves were harvested 3 days after infiltration, and protein isolation was conducted as previously described [56].

#### Heterologous protein production and purification for in vitro experiments

Bacteria expressing heterologous proteins were grown in auto-induction media at 37 °C and transferred to 16 °C overnight upon induction. Cell pellets were resuspended in buffer A1 (50 mM Tris-HCl, pH 8, 500 mM NaCl, 50 mM glycine, 5% [v/v] glycerol, 20 mM imidazole, and EDTA free protease inhibitor). The cells were lysed by sonication and subsequently spun to produce the clear lysate. A single Ni2+-NTA capture step was followed by gel filtration onto a Superdex 75 26/600 gel filtration column pre-equilibrated in buffer A4 (20 mM HEPES, pH 7.5, 150 mM NaCl). The fractions containing His-tagged ATG8 protein of interest were pooled and concentrated as appropriate, and the final concentration was judged by absorbance at 280 nm (using a calculated molar extinction coefficient of each protein). The purity of proteins was judged by running 16% SDS-PAGE gels stained with instant blue. PexRD54 was purified as described previously [36].

### Protein–protein interaction studies

#### ITC

All calorimetry experiments were recorded using a MicroCal PEAQ-ITC (Malvern, UK). To test the interaction of ATG8 proteins with full-length PexRD54, experiments were carried out at 18 °C using 20 mM HEPES, pH 7.5, 500 mM NaCl buffer. The calorimetric cell was filled with 110 μM PexRD54 and titrated with 1.1 mM ATG8 protein. For the PexRD54 AIM peptide studies, all experiments were conducted at 25 °C using 20 mM HEPES, pH 7.5, 150 mM NaCl buffer. The calorimetric cell was filled with 110 μM of ATG8 titrated with 1.1 mM of peptide. For each ITC run, a single injection of 0.5 μL of ligand was followed by 19 injections of 2 μL each. Injections were made at 120-second intervals with a stirring speed of 750 rpm. The raw titration data were integrated and fitted to a one-site binding model using the built-in software of MicroCal PEAQ ITC.

#### Co-IP and immunoblot analyses

The Co-IP protocol was described previously [56]. IP was performed by affinity chromatography with GFP_Trap_A beads (Chromotek). Proteins were eluted from the beads by heating 10 minutes at 70 °C. Proteins were separated by SDS-PAGE and were transferred onto a polyvinylidene diflouride membrane using a Trans-Blot turbo transfer system (Bio-Rad, Munich). The membrane was blocked with 5% milk in Tris-buffered saline and Tween 20. GFP detection was performed in a single step by a GFP (B2):sc-9996 horseradish peroxidase (HRP)-conjugated antibody (Santa Cruz Biotechnology, Santa Cruz, CA); RFP detection was performed with a rat anti-RFP 5F8 antibody (Chromotek, Munich) and an HRP-conjugated anti-rat antibody. Membrane imaging was carried out with an ImageQuant LAS 4000 luminescent imager (GE Healthcare Life Sciences, Piscataway, NJ). Instant Blue (Expedeon, Cambridge) staining of rubisco was used as a loading control.

### MS

#### (i) ATG8 interactome

Sample preparation for MS. Following the protein purification and washing steps, beads were resuspended in 100 mM ammonium bicarbonate (Fluka 09830-500G). To digest the immunoprecipitated proteins, 400 ng Lys-C (Wako PEF 7041) was added to the beads, followed by incubation for 4 hours at 37 °C with agitation. The supernatant was then reduced in 0.6 mM Tris 2-carboxyethyl phosphine hydrochloride (TCEP-HCl; Sigma 646547–10 × 1 mL) for 30 minutes at 60 °C followed by alkylation in 4 mM methyl methanethiosulfonate (MMTS; Fluka 64306) for 30 minutes at room temperature in the dark. To further digest the peptides, 400 ng Trypsin (Trypsin Gold Promega V5280) was added before incubation overnight at 37 °C. The digestion reaction was stopped by addition of trifluoroacetic acid (TFA; Aldrich T63002) to a final concentration of 1%.

NanoLC-MS analysis. LC-MS/MS analysis was performed with an UltiMate 3000 RSLC nano system (Thermo Fisher Scientific, Amsterdam, the Netherlands) coupled to a Q Exactive HF-X mass spectrometer (Thermo Fisher Scientific, Bremen, Germany) equipped with a Proxeon nanospray source (Thermo Fisher Scientific, Odense, Denmark). Peptides were loaded onto a trap column (Thermo Fisher Scientific, Amsterdam, the Netherlands, PepMap C18, 5 mm × 300 μm ID, 5-μm particles, 100-Å pore size), which was subsequently switched in line with the analytical column (Thermo Fisher Scientific, Amsterdam, the Netherlands, PepMap C18, 500 mm × 75 μm ID, 2 μm, 100 Å). Peptides were eluted using a binary 3-hour gradient and a flow rate of 230 nL/minute. The gradient used starts with the mobile phases, 98% A (water/formic acid, 99.9/0.1, v/v) and 2% B (water/acetonitrile/formic acid, 19.92/80/0.08, v/v/v), increasing to 35% B over the next 180 minutes, followed by a gradient in 5 minutes to 90% B, staying there for 5 minutes before decreasing in 2 minutes back to the gradient 98% A and 2% B for equilibration at 30 °C.

The Q Exactive HF-X mass spectrometer was operated in data-dependent mode using a full scan (m/z range 350–1,500, nominal resolution of 60,000, target value 1 × 10^6^), followed by MS/MS scans of the 10 most abundant ions. MS/MS spectra were acquired using normalized collision energy of 28, isolation width of 1.0 m/z, resolution of 30.000, and a target value set to 1 × 10^5^. Precursor ions were selected for fragmentation (exclude charge state 1, 7, 8, >8) and put on a dynamic exclusion list for 60 seconds. The minimum automatic gain control target was set to 5 × 10^3^ and intensity threshold was calculated to be 4.8 × 10^4^. The peptide match feature was set to the preferred mode, and the feature to exclude isotopes was enabled.

Data processing and peptide identification. For peptide identification, the RAW files were loaded into Proteome Discoverer (version 2.1.0.81, Thermo Scientific). Peptide identification was performed by searching the *N*. *benthamiana* genome database called Nicotiana_Benthamiana_Nbv6trPAplusSGNUniq_20170808 (398,682 sequences; 137,880,484 residues), supplemented with common contaminants using MSAmanda v2.1.5.9849, Engine version v2.0.0.9849 [57]. For this search, the peptide mass tolerance was set to ±5 ppm and the fragment mass tolerance to 15 ppm. The threshold for the number of missed trypsin cleavages was set to two. The result was filtered to 1% protein false discovery rate using the Percolator algorithm integrated in Thermo Proteome Discoverer. A sub-database was generated for further processing.

The RAW files were then searched against the sub-database (36,152 sequences; 16,892,506 residues). The following search parameters were used. Beta-methylthiolation on cysteine was set as a fixed modification. Variable modifications were set as oxidation on methionine; deamidation on asparagine and glutamine; acetylation on lysine; phosphorylation on serine, threonine and tyrosine; methylation and di-methylation on lysine and arginine; tri-methylation on lysine; and ubiquitination on lysine. Monoisotopic masses were searched within unrestricted protein masses for tryptic enzymatic specificity. The peptide mass tolerance was set to ±5 ppm and the fragment mass tolerance to ±15 ppm. The maximal number of missed cleavages was set to two. The result was filtered to 1% peptide false discovery rate using the Percolator algorithm integrated in Thermo Proteome Discoverer. The prediction of posttranslational modification sites within the peptides was performed with the tool ptmRS, based on the tool phosphoRS [58]. Peptide areas were quantified using the in-house-developed tool APQuant. The MS proteomics data have been deposited to the ProteomeXchange Consortium via the PRIDE [59,60] partner repository with the dataset identifier PXD011226.

Data filtering. For each assayed construct, the peptide-to-spectrum match (PSM) values were averaged between replicates, and then the dataset was filtered to remove any proteins that showed a PSM value of >10 with the empty vector control, as well as any proteins in which none of the ATG8-GFP fusions exhibited an average PSM value >10. After this basic filtering, the dataset was further filtered such that, for all interactors with an EV average PSM value of >4, at least one of the ATG8-GFP fusions had to exhibit >3× the EV PSM value (e.g., for EV average PSM = 9.5, one ATG8 > 28.5). This resulted in a final list of 621 proteins (S1 Table).

Network analysis. For each interactor in the dataset, the closest *A*. *thaliana* homolog was predicted using BLAST, and the GO annotations were obtained using Blast2GO [61]. The GO annotations were used to reduce the complexity of the final interactome presented in S1 Table. The interactors were sorted by cellular compartment or biological process, respectively, and then collapsed based on shared annotations. The number of interactors in each shared annotation group was recorded, and the average PSM values were calculated for each group for every ATG8; the resulting tables were imported into Cytoscape [62] to make the network figures. The former values were used to scale the node sizes across all network representations, and the latter values were used to weight the edges for each individual ATG8-group connection.

#### (ii) ATG8-4-S3 mutant analysis

Sample preparation for MS. Immunoprecipitated protein samples were separated by SDS-PAGE (4%–20% gradient gel, Biorad). After staining with Coomassie brilliant Blue G-250 (SimplyBlue Safe stain, Invitrogen), the proteins were cut out and the gel slices were destained in 50% acetonitrile. Reduction and alkylation reactions were performed by incubating the gel slices for 45 minutes in 10 mM DTT, followed by 30 minutes in the dark in 55 mM chloroacetamide. After washing with 25 mM ammonium bicarbonate, the slices were dehydrated in 100% acetonitrile. Gel slices were rehydrated with 50 mM ammonium bicarbonate and 5% acetonitrile containing 20 ng/μL trypsin (Pierce); digestion proceeded overnight at 37 °C. Tryptic peptides were sonicated from the gel in 5% formic acid and 50% acetonitrile, and the total extracts were evaporated until dry.

Data processing and peptide identification. IP-MS analysis of the GFP control and ATG8-GFP IP samples was done as previously described [63]. Briefly, LC-MS/MS analysis was performed with an Orbitrap Fusion Trihybrid mass spectrometer (Thermo Scientific) and a nanoflow-HPLC system (Dionex Ultimate3000, Thermo Scientific), described previously [63]. Peptide identification was performed by searching the in-house *N*. *benthamiana* database Nicotiana_Benthamiana_Nbv6trPAplusSGNUniq_20170808 (398,682 sequences; 137,880,484 residues) using Mascot (v 2.4.1 Matrix Science), allowing Trypsin peptide termini. Scaffold (v4; Proteome Software) was used to validate MS/MS-based peptide and protein identifications and annotate spectra. A search criterion of having a minimum of two peptides with MASCOT ion scores above 20 and with 95% peptide identity was used, and selected spectra were manually inspected before acceptance. The MS proteomics data have been deposited into the ProteomeXchange Consortium via the PRIDE [59,60] partner repository with the dataset identifier PXD011484.

Data filtering. The peptide count values were first normalized to the peptide counts for GFP in each sample to adjust for varying expression levels. Then, after averaging the replicate values for each assayed construct, the dataset was filtered to remove any proteins that showed a peptide count of >6 with the empty vector control, as well as any proteins in which none of the ATG8-GFP fusions exhibited a peptide count of >10. This resulted in a list of 496 proteins. This dataset was further filtered by removing proteins that showed extreme unevenness among replicates, resulting in a final ATG8-4-S3 dataset of 291 proteins amenable to statistical analysis (S16 Fig).

### Protein confirmation

#### Intact MS for accurate mass determination

LC-MS analysis, performed using standard procedures within the John Innes Centre proteomics facility, confirmed that each of the heterologously expressed proteins had the molecular mass as expected from the expressed sequence (S10, S11 and S13 Figs).

### Structural homology modelling

Due to high sequence identity, ATG8-2.2 was used as a template to generate a homology model of ATG8-4. The amino acid sequence of ATG8-4 was submitted to Protein Homology Recognition Engine V2.0 (Phyre^2^) for modelling [64]. The coordinates of ATG8-2.2 structure (5L83) were retrieved from the Protein Data Bank (PDB) and assigned as modelling template by using Phyre^2^ Expert Mode. The resulting model of ATG8-4 comprised amino acids Thr-4 to Glu-112 and was illustrated in CCP4MG software [65].

## Supporting information

S1 Fig Orthologous relationships between solanaceous ATG8 isoforms. A more detailed view of Fig 1a. Unrooted maximum-likelihood phylogenetic tree of 29 ATG8 homologs, with clades marked on the right and colors indicating plant species. The tree was calculated in MEGA7 [38] from a 369-nucleotide alignment (MUSCLE [39], codon-based). The bootstrap supports of the major nodes are indicated. The scale bar indicates the evolutionary distance based on nucleotide substitution rate. ATG8, autophagy-related protein 8.
(TIF)

S2 Fig Orthologous relationships between *S*. *tuberosum* and *N*. *benthamiana* ATG8 isoforms. (A-D) Alignments of *S*. *tuberosum* and *N*. *benthamiana* ATG8s by clade (MUSCLE [39]), visualized with Jalview. *S*. *tuberosum* ATG8s are named as in S1 Fig; (B) only the *S*. *tuberosum* ATG8-2.2 is shown for the clade 2 alignment, as both ATG8-2.1 and ATG8-2.2 have the same amino acid sequence. ATG8, autophagy-related protein 8.
(TIF)

S3 Fig Sequence diversity among potato ATG8 isoforms. Alignment of all *S*. *tuberosum* ATG8s (MUSCLE [39], visualized with Jalview, with the protein model above corresponding to the ATG8-2.2 structure). ATG8s are named as in S1 Fig; only ATG8-2.2 is included in the alignment, as both ATG8-2.1 and ATG8-2.2 have the same amino acid sequence. The boundaries of the Swap 3 region are indicated by brackets, and the amino acids mutated in the ATG8-4 point mutants are marked by asterisks (*). ATG8, autophagy-related protein 8.
(TIF)

S4 Fig ATG8 interactome data are reproducible across replicates. The PSM values for the two replicates of each ATG8 isoform in the interactome dataset (621 interactors) were plotted in a pairwise fashion with a line of best fit, showing reproducibility across the replicates. The *R*^2^ values for each correlation are reported for each pair of replicates. ATG8, autophagy-related protein 8; PSM, peptide-to-spectrum match.
(TIF)

S5 Fig Solanaceous ATG8 isoforms have distinct protein interaction profiles. The average PSM values for each ATG8 isoform in the interactome dataset (621 interactors) were used to generate a correlation matrix, showing distinct interaction profiles for each ATG8, with varying degrees of overlap. ATG8, autophagy-related protein 8; PSM, peptide-to-spectrum match.
(TIF)

S6 Fig Graphical abstract for ATG8 interactome network representations. For both Fig 1c and S7 Fig, nodes are scaled to the number of interactors present in each respective GO annotation group, and edges are weighted to the average PSM values for all of the proteins in that GO annotation group for each ATG8. Nodes are labelled where the average PSM values are most differential when compared with other ATG8s. ATG8, autophagy-related protein 8; PSM, peptide-to-spectrum match.
(TIF)

S7 Fig Network representation of the interactions between ATG8s and protein groups defined by biological process GO annotations. For each interactor in the dataset, the closest *A*. *thaliana* homolog was predicted using BLAST, and the GO annotations were obtained using Blast2GO [60]. Proteins were grouped based on the cellular compartment terms, and a subset of groups were chosen for representation. The sizes of the nodes are scaled to the number of interactors in each respective group, and the edges are weighed to the average PSM values for all the interactors in each respective group for each ATG8. Nodes are labelled where the average PSM value is most differential compared with the other ATG8s. Nodes shaded in gray exhibit similar average PSM values between all ATG8s, and the labels for these are included in the gray box. S6 Fig provides a graphical figure legend. ATG8, autophagy-related protein 8; GO, gene ontology; PSM, peptide-to-spectrum match.
(TIF)

S8 Fig Network representation of interaction between potato ATG8s and endogenous *N*. *benthamiana* ATG8s. (A) Network representation of the interactions between potato ATG8s and endogenous *N*. *benthamiana* ATG8s. The edge widths are weighted to the GFP normalized peptide counts shown in (B). The spatial relationships between the ATG8s are approximately scaled to amino acid sequence identity, with more sequence-related ATG8s clustering together, using Cytoscape [61]. The four *N*. *benthamiana* ATG8s present in the ATG8 interactome dataset—labelled here as NbATG8-1– NbATG8-4—are correspondingly labelled in S1 Table and S1 Fig, for reference. ATG8, autophagy-related protein 8; GFP, green fluorescent protein.
(TIF)

S9 Fig Significant level of overlap between the *N*. *benthamiana* ATG8 interactome and the human ATG8 interactome from Behrends and colleagues (2010) [22]. (Left) Graphical representation of the related proteins shared between the *N*. *benthamiana* ATG8 interactome (621 proteins) and the human ATG8 interactome from Behrends and colleagues (776 proteins), with the amount of overlap between the interactome circles scaled to the percent of *N*. *benthamiana* ATG8 interactors shared. The number of *N*. *benthamiana* ATG8 interactors with a related protein in the human ATG8 interactome is listed above the gray arrow, to the left, whereas the number of human ATG8 interactors with a related protein in the *N*. *benthamiana* ATG8 interactome are listed above the gray arrow, to the right. The discrepancy between these numbers is due to the existence of paralogous proteins in *N*. *benthamiana* or potential false duplications within the *N*. *benthamiana* proteome. (Right) Analogous graphical representation of the related proteins shared between three random sets of proteins (621 proteins each), separately, and the human ATG8 interactome from Behrends and colleagues (776 proteins). ATG8, autophagy-related protein 8.
(TIF)

S10 Fig (A) Coomassie-Blue-stained SDS/PAGE gel showing purified ATG8 isoforms used in in vitro binding studies. (B) Intact masses for ATG8 isoforms expressed and purified in this study. (C) Table summarizing the thermodynamic and kinetic data that were extracted for each ITC run between the PexRD54 AIM peptide and ATG8 isoforms. (D) Second replicate of ITC measuring the interaction between the PexRD54 AIM peptide and ATG8 isoforms. The top panels show heat differences upon injection of ligands and lower panels show integrated heats of injection (•) and the best fit (solid line) to a single site binding model using MicroCal PEAQ-ITC analysis software. AIM, ATG8-interacting motif; ATG8, autophagy-related protein 8; ITC, isothermal titration calorimetry; SDS/PAGE, sodium dodecyl sulfate/polyacrylamide gel electrophoresis.
(TIF)

S11 Fig (A) Coomassie-Blue-stained SDS/PAGE gel showing purified ATG8-4-S1 and ATG8-4-S3 used in in vitro binding studies. (B) Intact masses for ATG8 swaps (ATG8-4-S1 and ATG8-4-S3) and PexRD54 expressed and purified in this study. (C) Table summarizing the thermodynamic and kinetic data that were extracted for each ITC run between the PexRD54 full-length, PexRD54 AIM peptide, and ATG8 swaps. (C) Replicates of ITC measuring the interaction between ATG8 swaps and the PexRD54 AIM peptide (left) and full-length protein (right). The top panels show heat differences upon injection of ligands and lower panels show integrated heats of injection (•) and the best fit (solid line) to a single site binding model using MicroCal PEAQ-ITC analysis software. ATG8, autophagy-related protein 8; ITC, isothermal titration calorimetry; SDS/PAGE, sodium dodecyl sulfate/polyacrylamide gel electrophoresis.
(TIF)

S12 Fig Schematic representation of (A) ATG8-4 and (B) ATG8-2.2 with PexRD54 AIM peptide in the binding cavity. The molecular surface of each ATG8 that contacts the AIM peptide is shown in magenta. The AIM peptide is shown as a stick representation in each structure, with residues labelled. α-helices, β-strands, and N and C termini of ATG8-4 and ATG8-2.2 are labelled. AIM, ATG8-interacting motif; ATG8, autophagy-related protein 8.
(TIF)

S13 Fig (A) Coomassie-stained SDS-PAGE showing purified ATG8-4-V32I. (B) Identity of ATG8-4-V32I was confirmed by measuring intact mass using MS. (C) Table summarizing the thermodynamic and kinetic data that were extracted for each ITC run between the PexRD54 full-length, PexRD54 AIM peptide, and ATG8-4-V32I. (D) Second replicate of the ITC trace showing interaction between ATG8-4-V32I and PexRD54 AIM peptide. (E) Replicates of the ITC traces showing interaction between ATG8-4-V32I and the full-length PexRD54. AIM, ATG8-interacting motif; ATG8, autophagy-related protein 8; ITC, isothermal titration calorimetry; MS, mass spectrometry; SDS-PAGE, sodium dodecyl sulfate/polyacrylamide gel electrophoresis.
(TIF)

S14 Fig The first β-strand of ATG8 underpins interaction with plant proteins. For each interactor in the dataset, the average peptide count data for ATG8-2.2 (teal), ATG8-4 (light gray), and ATG8-4-S3 (green) were normalized to either ATG8-2.2 or ATG8-4 data based on the enrichment category being analyzed: (A) values for ATG8-2.2–enriched interactors were normalized to ATG8-2.2, (B) values for ATG8-4–enriched interactors were normalized to ATG8-4, and (C) values for common interactors were normalized to ATG8-2.2. For (A) ATG8-2.2–enriched interactors and (B) ATG8-4–enriched interactors, this highlights the difference in how ATG8-2.2 and ATG8-4 interact with each protein in the set and how the ATG8-4-S3 interactions compare. For (A) ATG8-2.2–enriched interactors, the asterisk (*) marks proteins that showed no statistical difference in their interaction with ATG8-4-S3 as compared with ATG8-2.2 (in Fig 6d, “(+) S3 enrichment”); for (B) ATG8-4 enriched interactors, the asterisk (*) marks proteins that showed no statistical difference in their interaction with ATG8-4-S3 as compared with ATG8-4. For (C) common interactors, the graph highlights the similarity in how ATG8-2.2, ATG8-4, and ATG8-4-S3 interact with each protein in the set; due to the lack of statistical difference, this feature is not marked. ATG8, autophagy-related protein 8.
(TIF)

S15 Fig The ATG8 region surrounding the first β-strand is responsible for discriminatory binding to potato Vps4. (A) Co-IP experiment between potato Vps4 and the Vps4 AIM mutant (Vps4^AIM^)—changing the AIM sequence of SDFEDL to SDAEDA—with ATG8-2.2. Vps4:3xMyc and Vps4^AIM^:3xMyc were transiently co-expressed with GFP:EV and GFP:ATG8-2.2, respectively. (b) Co-IP experiment between Vps4 and ATG8-2.2, ATG8-4, and ATG8-4-S3. Vps4:3xMyc was transiently co-expressed with GFP:ATG8-2.2, GFP:ATG8-4, and GFP:ATG8-4-S3. For (A-B), IPs were obtained with anti-GFP antiserum and total protein extracts were immunoblotted with appropriate antisera (listed on the right). Stars indicate expected band sizes. (C) Unrooted maximum-likelihood phylogenetic tree of orthologs of *Saccharomyces cerevisiae* (yeast) Vps4 from across select plant species, with the *N*. *benthamiana* Vps4 identified in the IP-MS experiment starred (*) and the *S*. *tuberosum* Vps4 tested in Co-IP experiments marked (“Vps4”). Colors indicate species, and bootstrap supports are noted when >0.7. The tree was calculated in MEGA7 [38] from a 448–amino acid alignment (MUSCLE [39], codon-based). The scale bar indicates the evolutionary distance based on amino acid substitution rate. (D) Alignment of Vps4 sequences included in the phylogenetic tree in (C), excluding the Nbv6.1trA11845 and Nbv6.1trP33573 sequences, which are almost sequence identical to NbS00008926g0010.1. The presence of a predicted AIM as determined by iLIR is marked with a red box [40]. AIM, ATG8-interacting motif; CoIP, co-immunoprecipitation; GFP:EV, green fluorescent protein empty vector; IP, immunoprecipitate; IP-MS, immunoprecipitation followed by mass spectrometry; Vps4, vacuolar protein sorting 4.
(TIF)

S16 Fig Normal distribution of comparative ATG8-4-S3 mutant analysis data. The standard deviation (stdev) versus mean is plotted for the GFP normalized peptide count data for three replicates of each construct tested in IP-MS, (A) ATG8-2.2, (B) ATG8-4, and (C) ATG8-4-S3, showing a normal distribution in each. (D) A histogram of ANOVA *p*-values showing the high level of significance within the dataset. ATG8, autophagy-related protein 8; GFP, green fluorescent protein; IP-MS, immunoprecipitation followed by mass spectrometry.
(TIF)

S1 Table ATG8 interactome. For each *N*. *benthamiana* interactor in the dataset (621 proteins), putative AIMs were predicted using iLIR [40], the closest *A*. *thaliana* and *M*. *polymorpha* homologs were predicted using BLAST, and the AIMs in these homologs were again predicted using iLIR. Each interactor is thus described, by column: *N*. *benthamiana* accession (“Nb”), protein identification (“Nb_protein_ID”), and number of putative AIMs (“Nb_AIMs”); the *A*. *thaliana* homolog accession number (“At”), BLAST %identity (“At_%ID”), BLAST Expect (E) value (“At_evalue”), protein identification (“At_protein_ID”), number of putative *N*. *benthamiana* AIMs conserved (“At_conserved_AIMs”), and GO annotations (“At_compartment” and “At_process_function”) determined using Blast2GO [61]; the *M*. *polymorpha* homolog accession number (“Mp”), BLAST %identity (“Mp_%ID”), BLAST E value (“Mp_evalue”), protein identification (“Mp_protein_ID”), and number of putative *N*. *benthamiana* AIMs conserved (“Mp_conserved_AIMs”). The related proteins within the Behrends and colleagues dataset, as summarized in S9 Fig, are listed for each relevant interactor (“human_gene_name”). In addition, the average PSM values for the two replicates for each ATG8 isoform, as well as EV, are appended. AIM, ATG8-interacting motif; ATG8, autophagy-related protein 8; EV, empty vector; GO, gene ontology; PSM, peptide-to-spectrum match.
(XLSX)

S2 Table Overlap between the ATG8 interactome and human ATG8 interactome from Behrends and colleagues [22]. The gene names of the candidate human ATG8 interactors from Behrends and colleagues (2010) (776 proteins) were used to retrieve the corresponding protein sequences from Ensembl and UniProt [66,67]. Proteins related to the human ATG8 interactors within the ATG8 interactome were determined by BLAST, with an expect (E) value cutoff of 1 × 10^−15^. The *N*. *benthamiana* interactors related to proteins in the human ATG8 interactome (297 proteins) are listed by accession (“Nb”), along with the *N*. *benthamiana* protein identification (“Nb_protein_ID”), the gene name of the related human ATG8 interactor (“Hs_gene_name”), the human protein identification (“Hs_protein_ID”), and the BLAST search results. ATG8, autophagy-related protein 8.
(XLSX)

S3 Table ATG8 interactome AIM sequences and conservation. For each *N*. *benthamiana* interactor in the IP-MS dataset, the putative AIMs were predicated using iLIR [40]. These putative AIMs are recorded by *N*. *benthamiana* accession (“Nb”), with the start (“Nb_start”) and end (“Nb_end”) points of the AIM included, as well as the sequence (“Nb_sequence”) and iLIR prediction score (“Nb_PSSM”). For *N*. *benthamiana* proteins with multiple predicted AIMs, all are included. For each protein, the closest *A*. *thaliana* (“At”) and *M*. *polymorpha* (“Mp”) homologs were also analysed for putative AIMs, and those conserved with *N*. *benthamiana* were recorded. For each conserved AIM, the conservation score (number of positions conserved) was defined (“At_conserved” and “Mp_conserved”), as well as the AIM start and end sites, sequence, and prediction score. AIM, ATG8-interacting motif; ATG8, autophagy-related protein 8; IP-MS, immunoprecipitation followed by mass spectrometry.
(XLSX)

S4 Table Overlap between ATG8 interactome and Swap3 interactome datasets. The ATG8 interactome (S1 Table) and Swap3 interactome (S4 Table) were cross-referenced by *N*. *benthamiana* accession (“Nb”) and protein description (“Nb_protein_ID”). Interactors defined as shared between the datasets were determined by matching accession numbers (green) or in a family-based manner by exact protein description (blue). ATG8, autophagy-related protein 8; Swap3, ATG8-4 chimera swap 3.
(XLSX)

S5 Table Comparative ATG8-4-S3 mutant analysis dataset. The *N*. *benthamiana* proteins in the dataset (291 proteins) were divided into enrichment categories based on whether they showed a significantly (*p* < 0.05) stronger interaction with ATG8-2.2 or ATG8-4, as determined by an ANOVA with a post hoc Tukey test; interactors that showed no significant difference in their interaction with either protein were categorized as “common” (“enrichment” column). This resulted in 178 interactors being defined as ATG8-2.2 enriched, 6 as ATG8-4 enriched, and 107 as common. For each ATG8-2.2 enriched interactor, we determined whether ATG8-4-S3 showed a significant (*p* < 0.05) difference in its interaction strength compared with ATG8-2.2, using an ANOVA with a post hoc Tukey test (“S3-2.2” column). Proteins that showed no statistical difference in their interaction with ATG8-4-S3 compared with ATG8-2.2 are categorized as “+”; those that showed a statistically weaker interaction are categorized as “−.” For each ATG8-4 specific interactor, it was determined whether ATG8-4-S3 showed a significant (*p* < 0.05) difference in its interaction strength compared with ATG8-4 using an ANOVA with a post hoc Tukey test (“S3-4” column). Proteins that showed no statistical difference in their interaction with ATG8-4-S3 compared with ATG8-4 are categorized as “+”; those that showed a statistically weaker interaction are categorized as “−.” In addition to this analysis, the same interactor descriptions from the ATG8 interactome (S1 Table) were included—including predicted AIMs, *A*. *thaliana* orthologs, GO annotations, *M*. *polymorpha* orthologs, and AIM conservation in both species—along with the averaged GFP normalized peptide count data for all constructs. AIM, ATG8-interacting motif; ATG8, autophagy-related protein 8; GFP, green fluorescent protein; GO, gene ontology.
(XLSX)

S6 Table Swap interactome AIM sequences and conservation. For each *N*. *benthamiana* interactor in the comparative ATG8-4-S3 mutant analysis, the putative AIMs were predicated using iLIR [40]. These putative AIMs are recorded by *N*. *benthamiana* accession (“Nb”), with the start (“Nb_start”) and end (“Nb_end”) points of the AIM included, as well as the sequence (“Nb_sequence”) and iLIR prediction score (“Nb_PSSM”). For *N*. *benthamiana* proteins with multiple predicted AIMs, all are included. For each protein, the closest *A*. *thaliana* (“At”) and *M*. *polymorpha* (“Mp”) homologs were also analyzed for putative AIMs, and those conserved with *N*. *benthamiana* were recorded. For each conserved AIM, the conservation score (number of positions conserved) was defined (“At_conserved” and “Mp_conserved”), as well as the AIM start and end sites, sequence, and prediction score. AIM, ATG8-interacting motif; ATG8, autophagy-related protein 8.
(XLSX)

S7 Table Primers used in this study (5′–3′). (XLSX)

## Abbreviations

AIM: ATG8-interacting motif
ATG: autophagy-related protein
ATG8: autophagy-related protein 8
Co-IP: co-immunoprecipitation
ER: endoplasmic reticulum
ESCRT: endosomal sorting complex required for transport
GIM: GABARAP interaction motif
GO: gene ontology
HRP: horseradish peroxidase
HSD: honestly significant difference
Ile-33: isoleucine 33
IP: immunoprecipitation
ITC: isothermal titration calorimetry
LIR: LC3-interacting region
MES: 2-(N-morpholine)-ethanesulfonic acid
MMTS: methyl methanethiosulfonate
MS: mass spectrometry
NbATG8-4: *N*. *benthamiana* ATG8-4
NSF: N-Ethylmaleimide-sensitive factor
PDB: Protein Data Bank
PiPexRD54: P. infestans PexRD54
PSM: peptide-to-spectrum match
SKD1: suppressor of K^+^ transport growth defect 1
SNARE: soluble NSF attachment protein receptor
TCEP-HCl: Tris 2-carboxyethyl phosphine hydrochloride
TFA: trifluoroacetic acid
Vps4: vacuolar protein sorting 4
