# Supplementary figures and images for: N-terminal β-strand underpins biochemical specialization of an ATG8 isoform
Source: PLoS Biol. 2019 Jul 22;17(7):e3000373. doi: 10.1371/journal.pbio.3000373 (PMC6675122; doi:10.1371/journal.pbio.3000373)

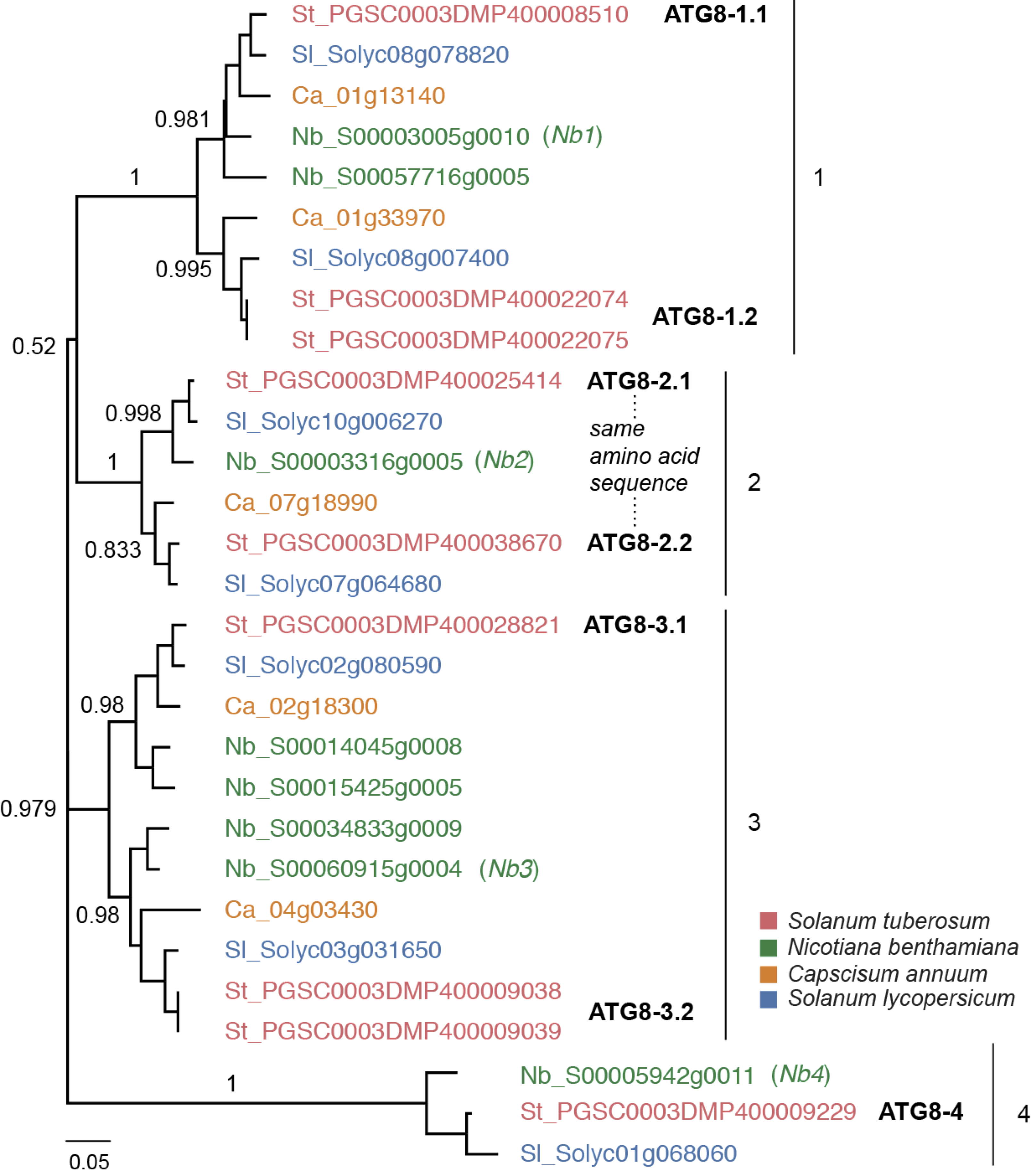

Supplement: S1 Fig — A more detailed view of Fig 1a. Unrooted maximum-likelihood phylogenetic tree of 29 ATG8 homologs, with clades marked on the right and colors indicating plant species. The tree was calculated in MEGA7 [38] from a 369-nucleotide alignment (MUSCLE [39], codon-based). The bootstrap supports of the major nodes are indicated. The scale bar indicates the evolutionary distance based on nucleotide substitution rate. ATG8, autophagy-related protein 8. (TIF) [file pbio.3000373.s001.tif]

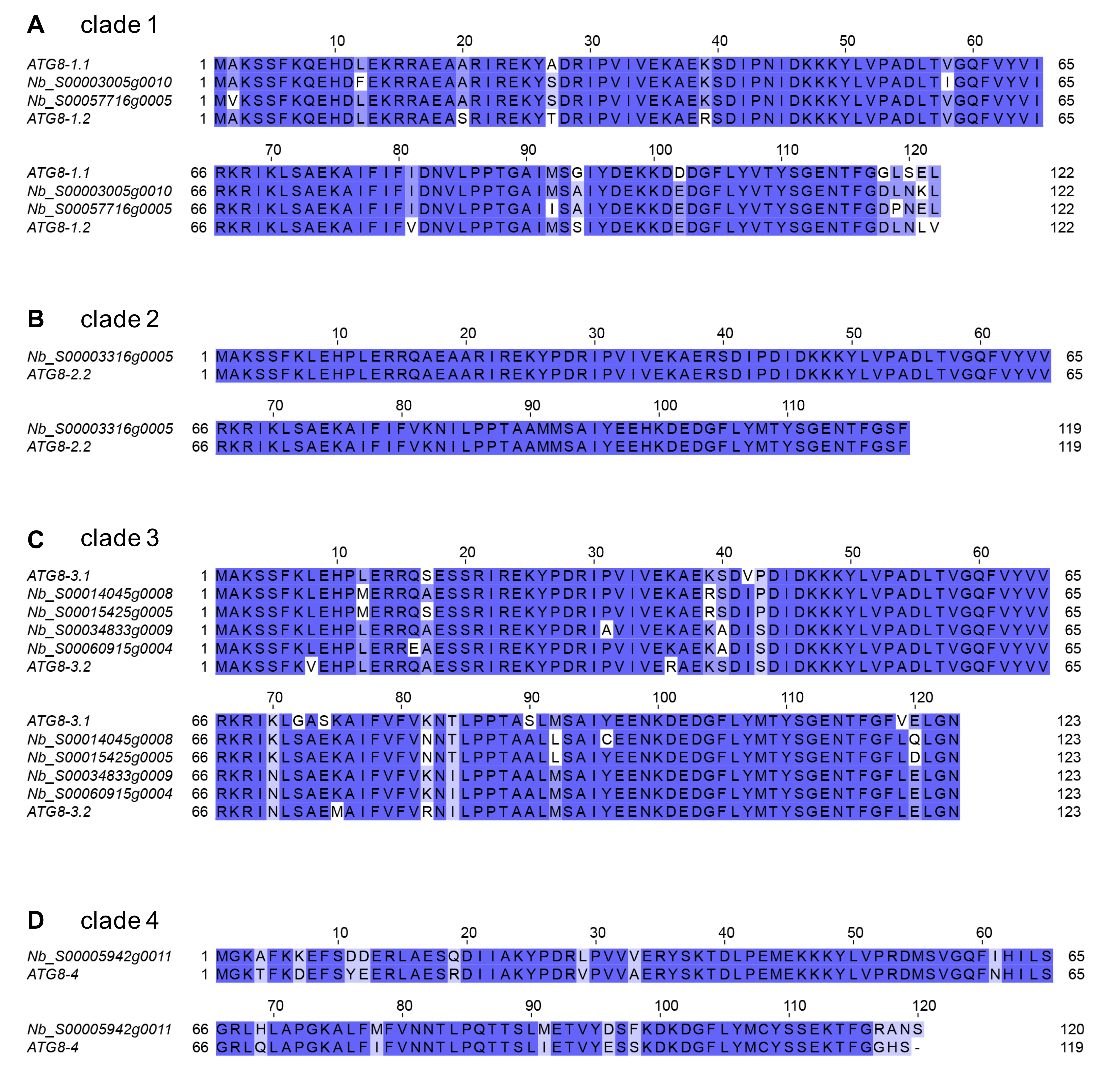

Supplement: S2 Fig — (A-D) Alignments of S. tuberosum and N. benthamiana ATG8s by clade (MUSCLE [39]), visualized with Jalview. S. tuberosum ATG8s are named as in S1 Fig; (B) only the S. tuberosum ATG8-2.2 is shown for the clade 2 alignment, as both ATG8-2.1 and ATG8-2.2 have the same amino acid sequence. ATG8, autophagy-related protein 8. (TIF) [file pbio.3000373.s002.tif]

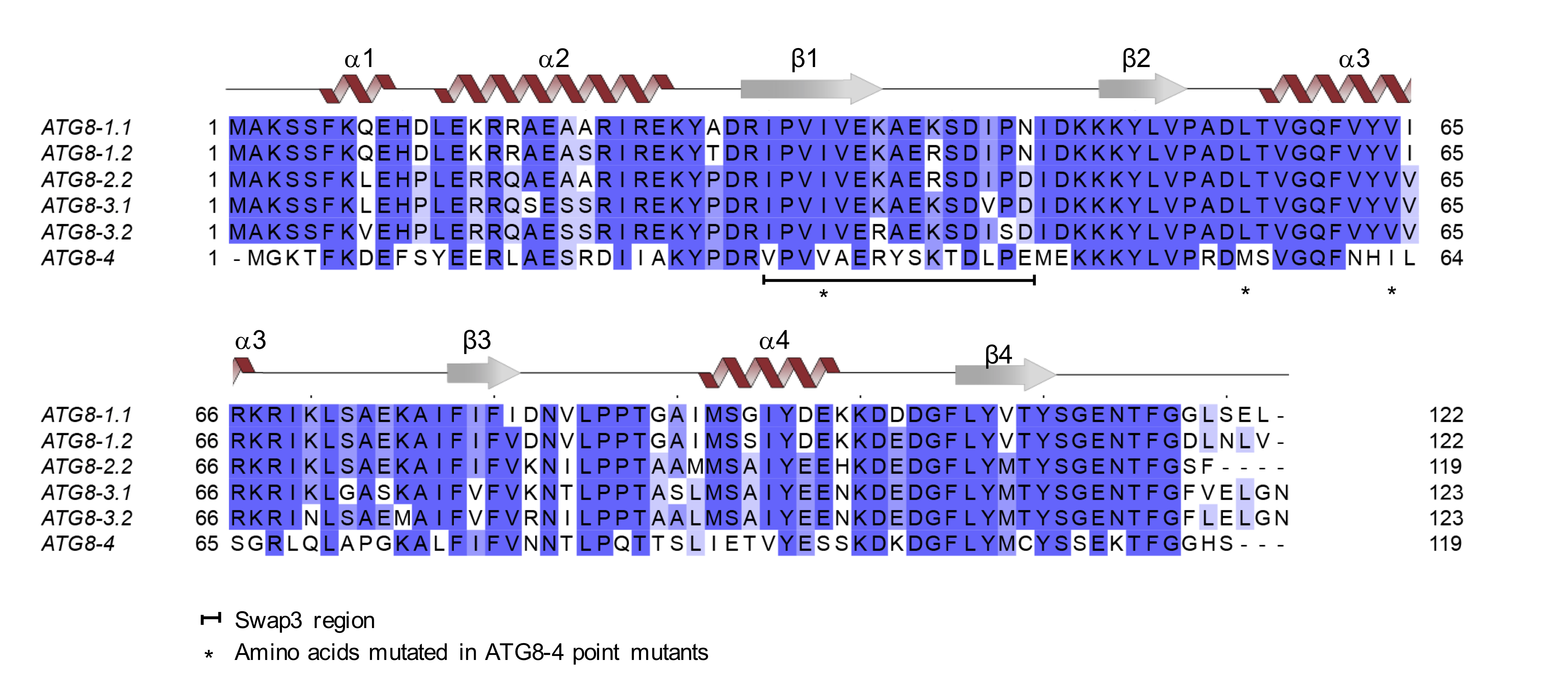

Supplement: S3 Fig — Alignment of all S. tuberosum ATG8s (MUSCLE [39], visualized with Jalview, with the protein model above corresponding to the ATG8-2.2 structure). ATG8s are named as in S1 Fig; only ATG8-2.2 is included in the alignment, as both ATG8-2.1 and ATG8-2.2 have the same amino acid sequence. The boundaries of the Swap 3 region are indicated by brackets, and the amino acids mutated in the ATG8-4 point mutants are marked by asterisks (*). ATG8, autophagy-related protein 8. (TIF) [file pbio.3000373.s003.tif]

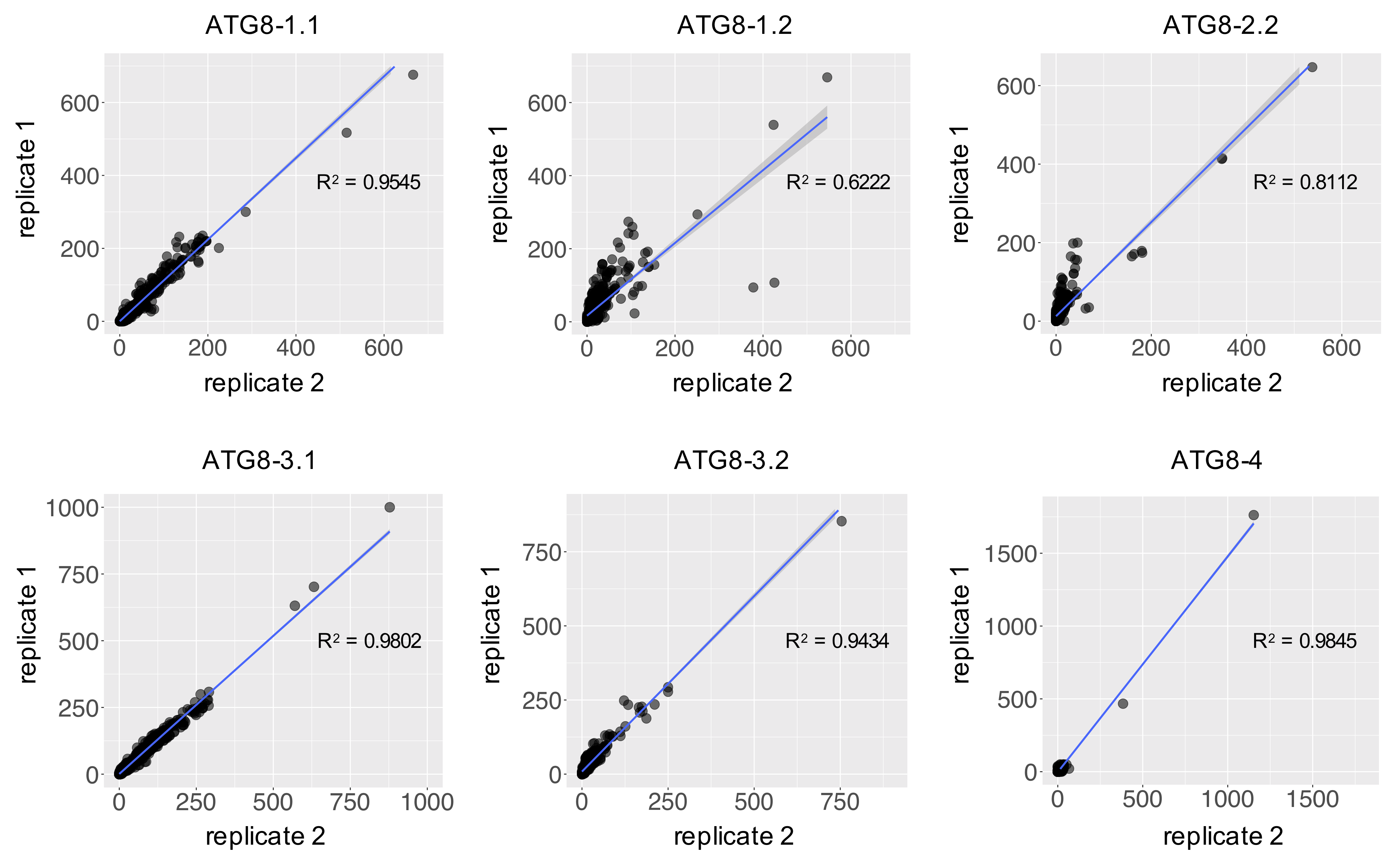

Supplement: S4 Fig — The PSM values for the two replicates of each ATG8 isoform in the interactome dataset (621 interactors) were plotted in a pairwise fashion with a line of best fit, showing reproducibility across the replicates. The R2 values for each correlation are reported for each pair of replicates. ATG8, autophagy-related protein 8; PSM, peptide-to-spectrum match. (TIF) [file pbio.3000373.s004.tif]

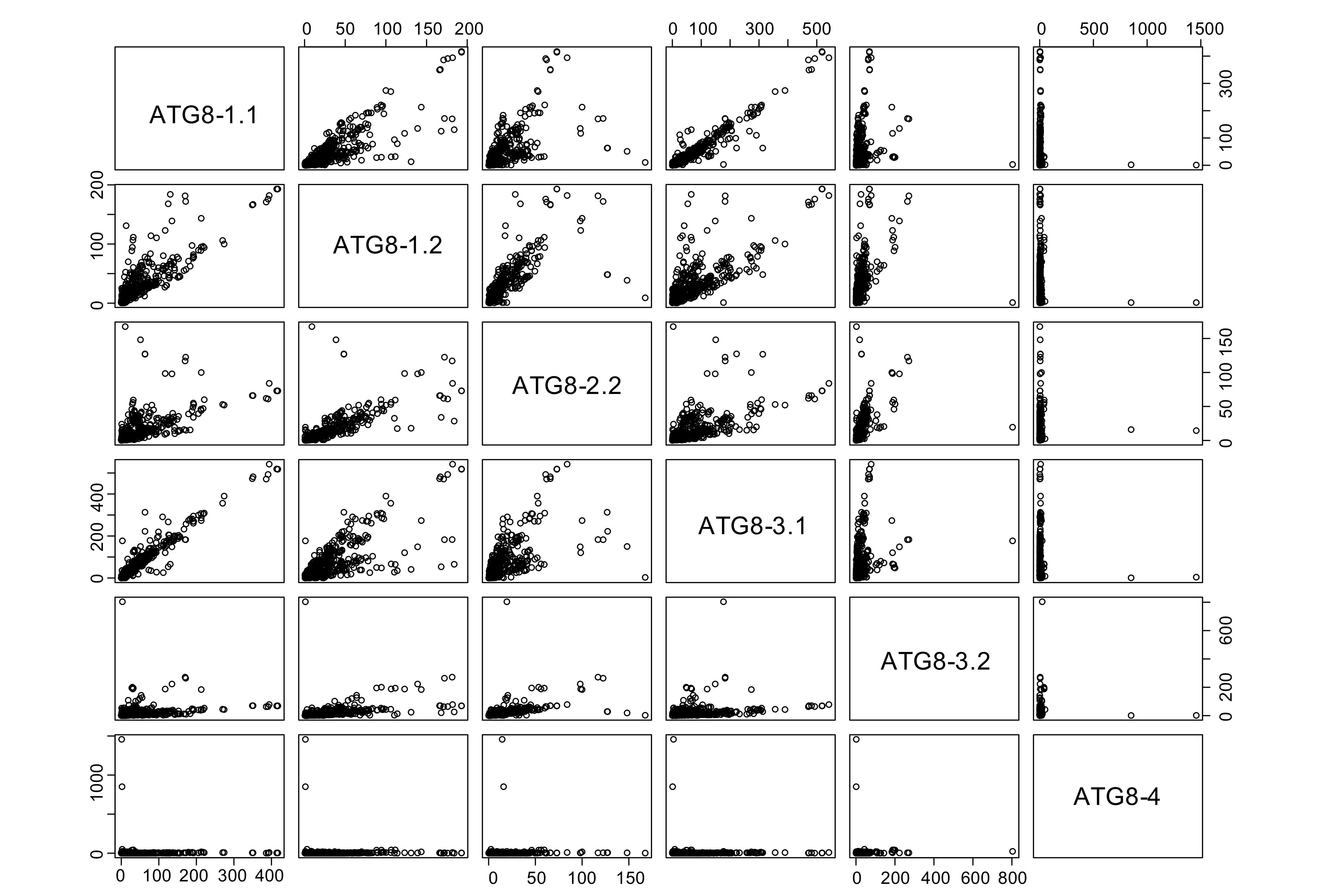

Supplement: S5 Fig — The average PSM values for each ATG8 isoform in the interactome dataset (621 interactors) were used to generate a correlation matrix, showing distinct interaction profiles for each ATG8, with varying degrees of overlap. ATG8, autophagy-related protein 8; PSM, peptide-to-spectrum match. (TIF) [file pbio.3000373.s005.tif]

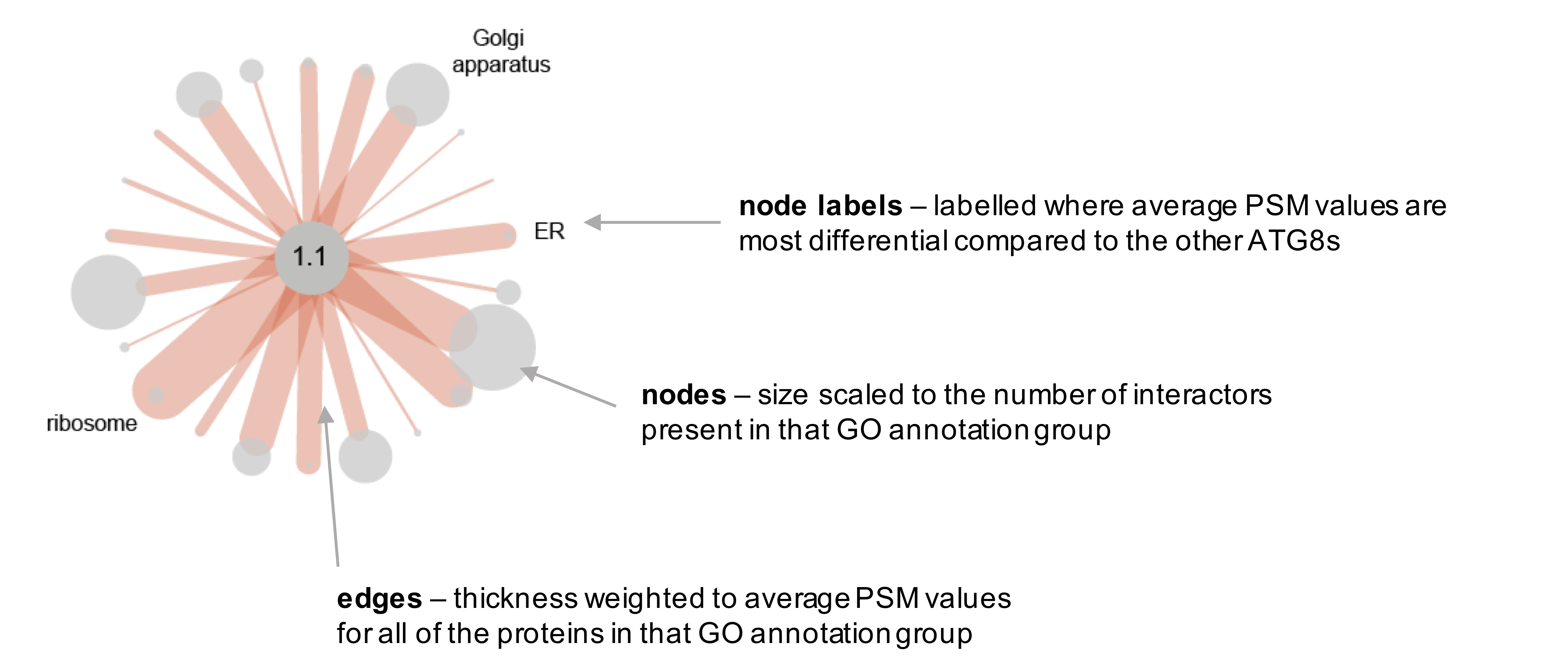

Supplement: S6 Fig — For both Fig 1c and S7 Fig, nodes are scaled to the number of interactors present in each respective GO annotation group, and edges are weighted to the average PSM values for all of the proteins in that GO annotation group for each ATG8. Nodes are labelled where the average PSM values are most differential when compared with other ATG8s. ATG8, autophagy-related protein 8; PSM, peptide-to-spectrum match. (TIF) [file pbio.3000373.s006.tif]

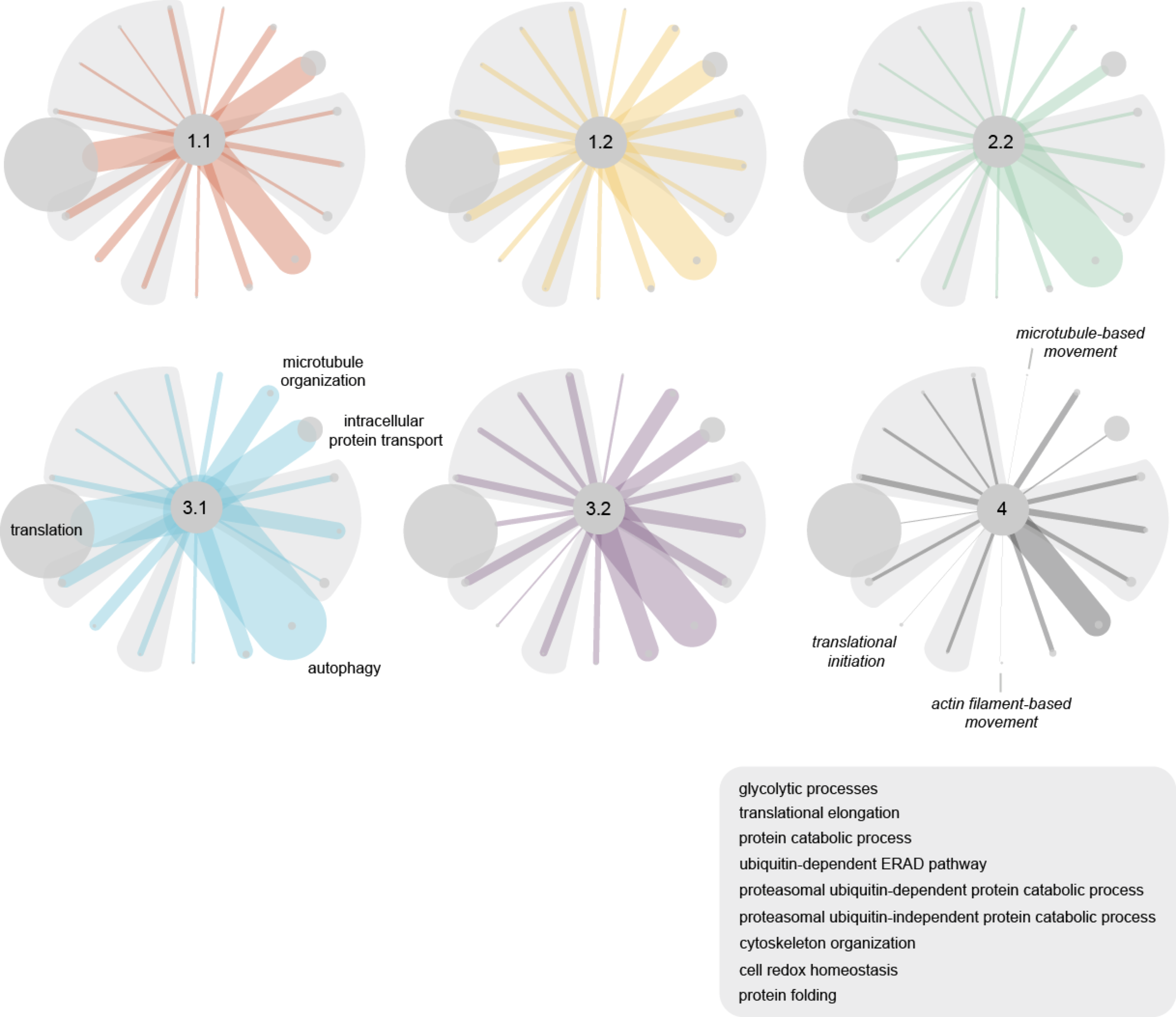

Supplement: S7 Fig — For each interactor in the dataset, the closest A. thaliana homolog was predicted using BLAST, and the GO annotations were obtained using Blast2GO [60]. Proteins were grouped based on the cellular compartment terms, and a subset of groups were chosen for representation. The sizes of the nodes are scaled to the number of interactors in each respective group, and the edges are weighed to the average PSM values for all the interactors in each respective group for each ATG8. Nodes are labelled where the average PSM value is most differential compared with the other ATG8s. Nodes shaded in gray exhibit similar average PSM values between all ATG8s, and the labels for these are included in the gray box. S6 Fig provides a graphical figure legend. ATG8, autophagy-related protein 8; GO, gene ontology; PSM, peptide-to-spectrum match. (TIF) [file pbio.3000373.s007.tif]

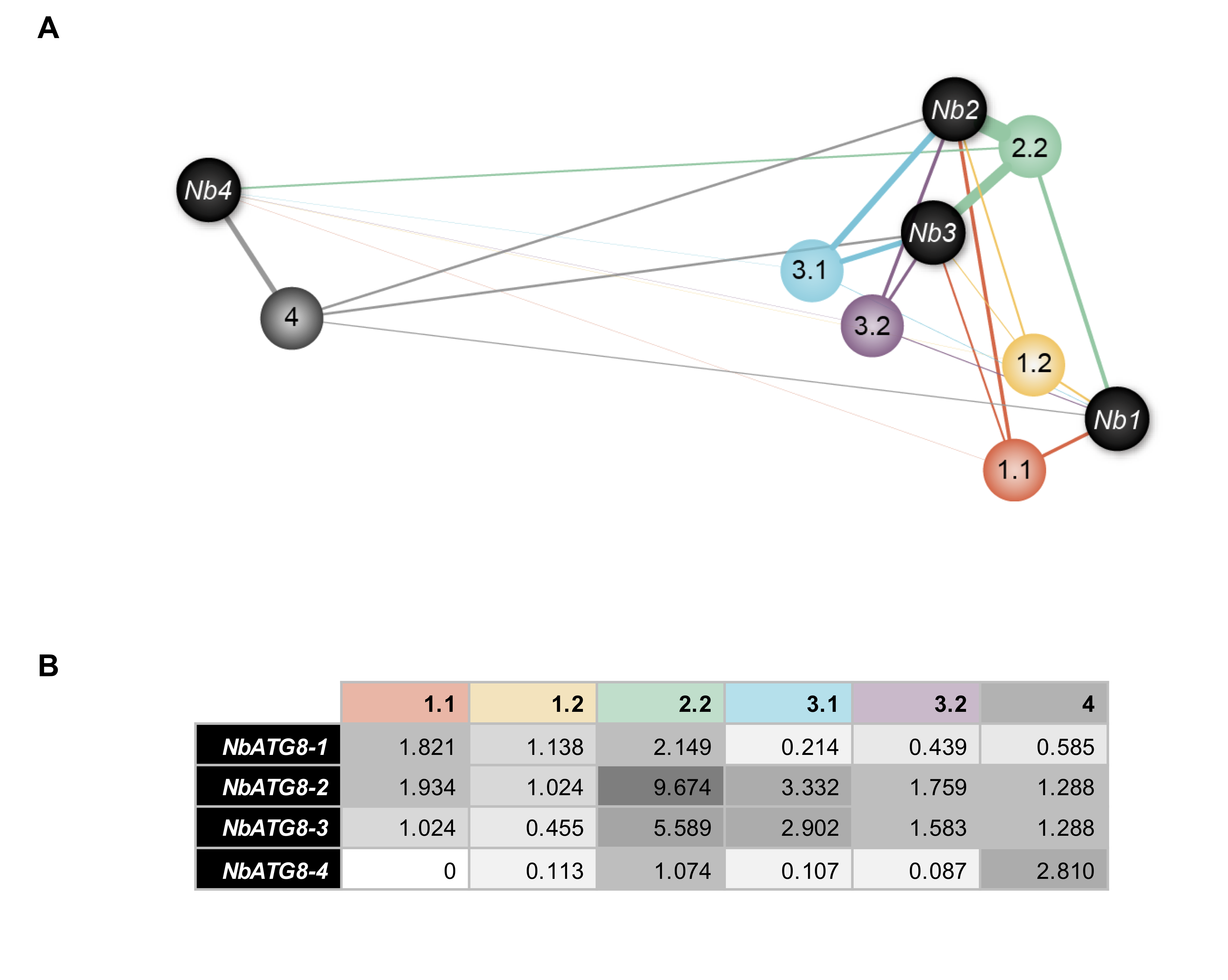

Supplement: S8 Fig — (A) Network representation of the interactions between potato ATG8s and endogenous N. benthamiana ATG8s. The edge widths are weighted to the GFP normalized peptide counts shown in (B). The spatial relationships between the ATG8s are approximately scaled to amino acid sequence identity, with more sequence-related ATG8s clustering together, using Cytoscape [61]. The four N. benthamiana ATG8s present in the ATG8 interactome dataset—labelled here as NbATG8-1– NbATG8-4—are correspondingly labelled in S1 Table and S1 Fig, for reference. ATG8, autophagy-related protein 8; GFP, green fluorescent protein. (TIF) [file pbio.3000373.s008.tif]

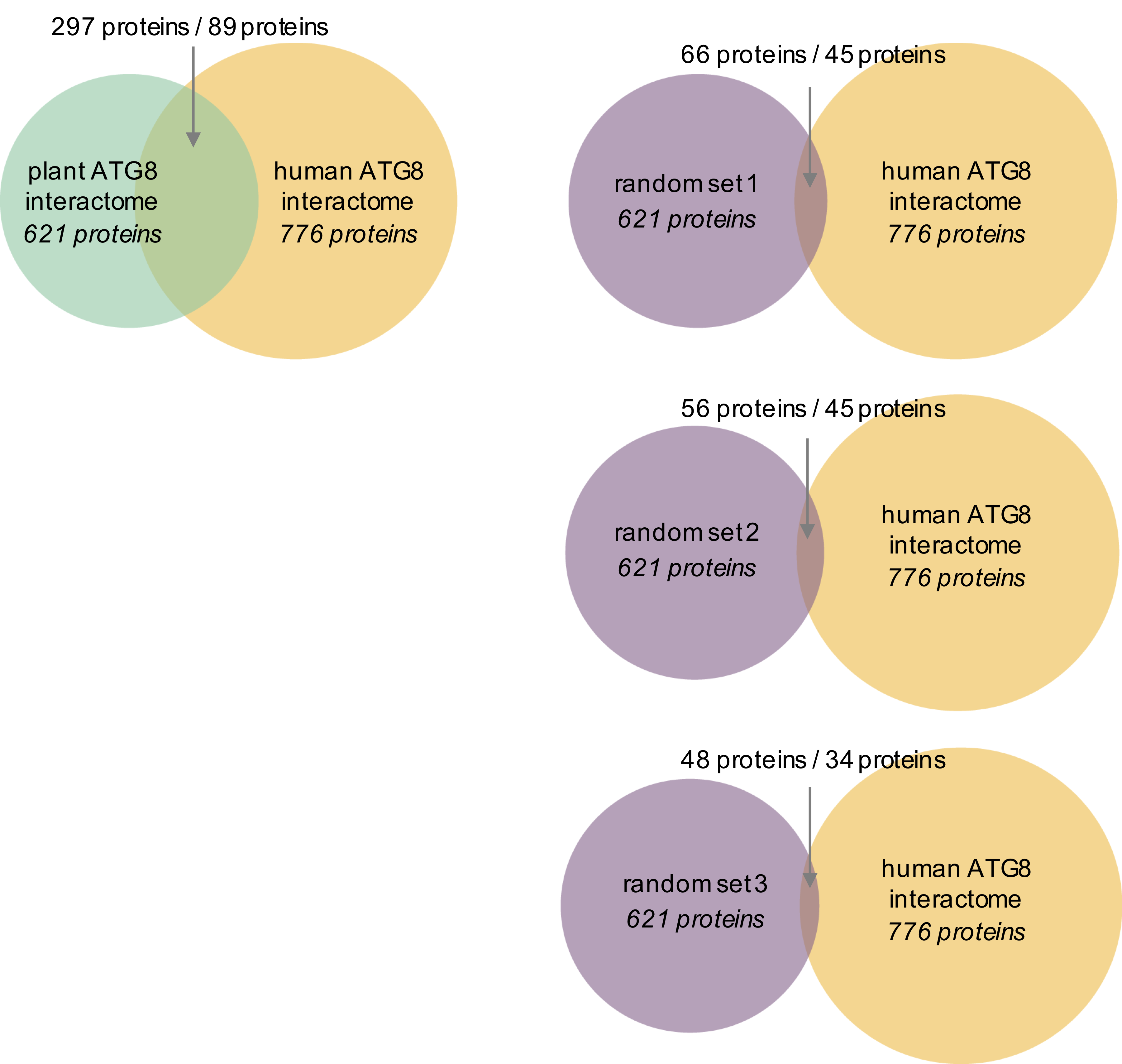

Supplement: S9 Fig — (Left) Graphical representation of the related proteins shared between the N. benthamiana ATG8 interactome (621 proteins) and the human ATG8 interactome from Behrends and colleagues (776 proteins), with the amount of overlap between the interactome circles scaled to the percent of N. benthamiana ATG8 interactors shared. The number of N. benthamiana ATG8 interactors with a related protein in the human ATG8 interactome is listed above the gray arrow, to the left, whereas the number of human ATG8 interactors with a related protein in the N. benthamiana ATG8 interactome are listed above the gray arrow, to the right. The discrepancy between these numbers is due to the existence of paralogous proteins in N. benthamiana or potential false duplications within the N. benthamiana proteome. (Right) Analogous graphical representation of the related proteins shared between three random sets of proteins (621 proteins each), separately, and the human ATG8 interactome from Behrends and colleagues (776 proteins). ATG8, autophagy-related protein 8. (TIF) [file pbio.3000373.s009.tif]

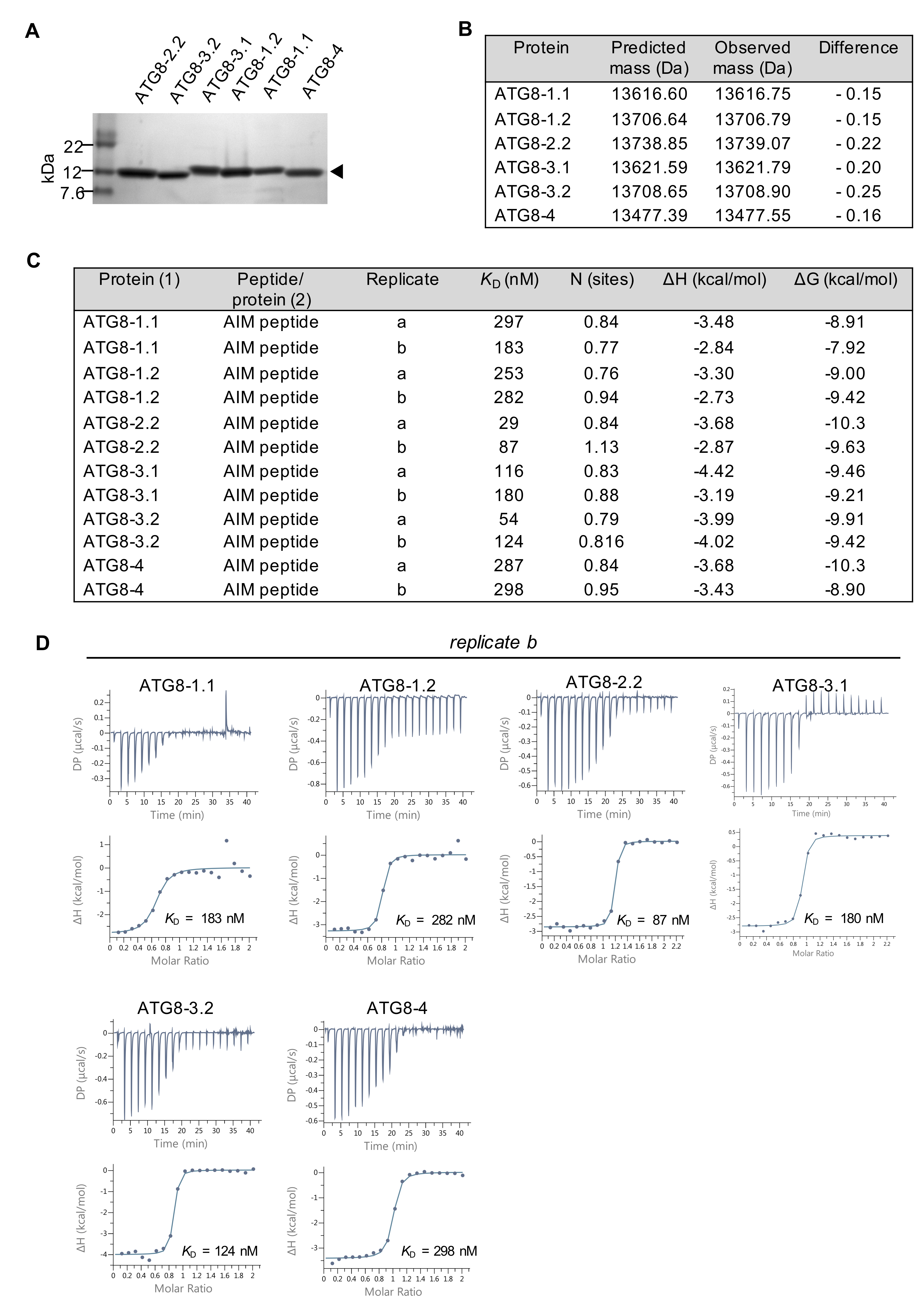

Supplement: S10 Fig — (A) Coomassie-Blue-stained SDS/PAGE gel showing purified ATG8 isoforms used in in vitro binding studies. (B) Intact masses for ATG8 isoforms expressed and purified in this study. (C) Table summarizing the thermodynamic and kinetic data that were extracted for each ITC run between the PexRD54 AIM peptide and ATG8 isoforms. (D) Second replicate of ITC measuring the interaction between the PexRD54 AIM peptide and ATG8 isoforms. The top panels show heat differences upon injection of ligands and lower panels show integrated heats of injection (•) and the best fit (solid line) to a single site binding model using MicroCal PEAQ-ITC analysis software. AIM, ATG8-interacting motif; ATG8, autophagy-related protein 8; ITC, isothermal titration calorimetry; SDS/PAGE, sodium dodecyl sulfate/polyacrylamide gel electrophoresis. (TIF) [file pbio.3000373.s010.tif]

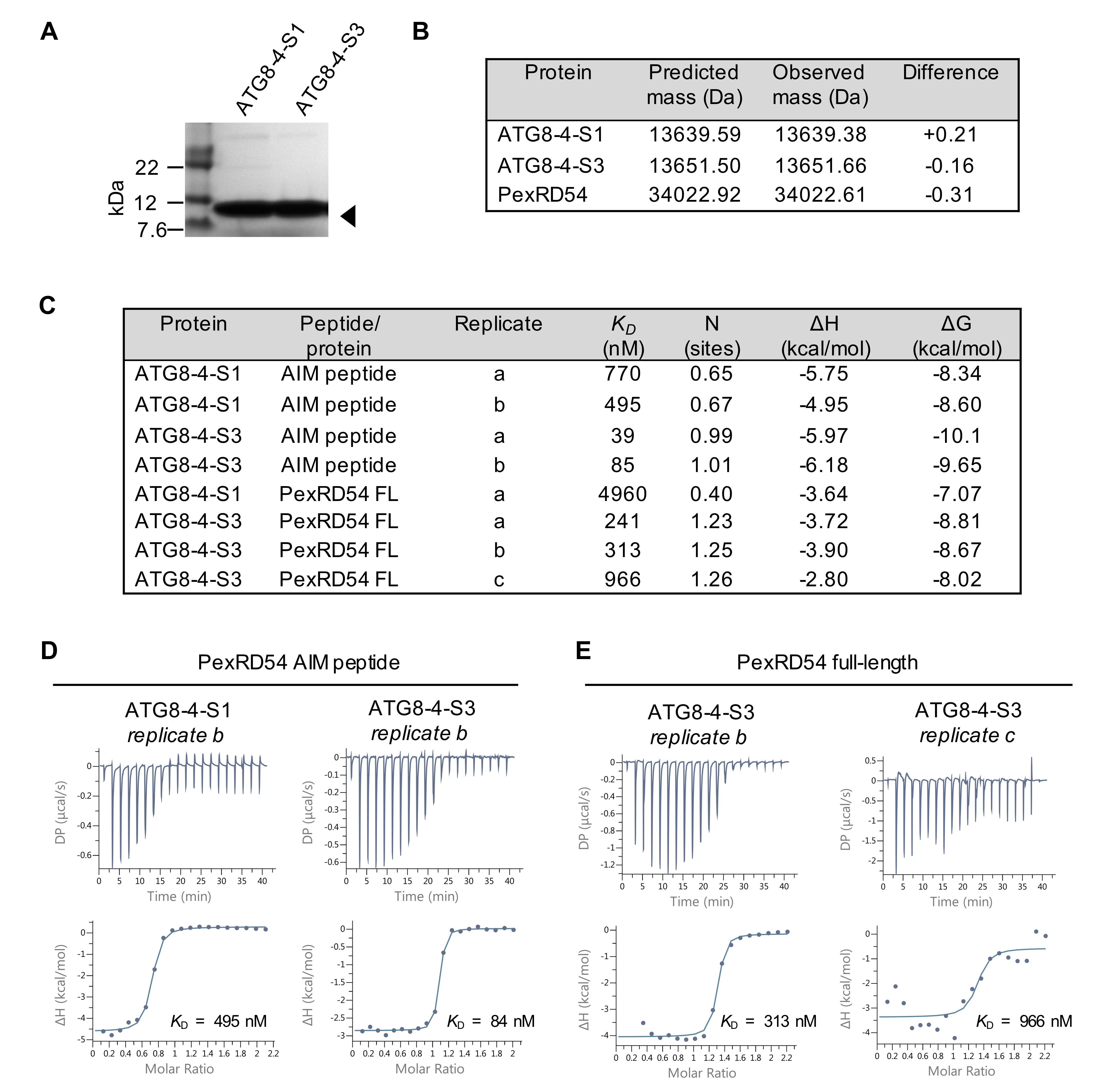

Supplement: S11 Fig — (A) Coomassie-Blue-stained SDS/PAGE gel showing purified ATG8-4-S1 and ATG8-4-S3 used in in vitro binding studies. (B) Intact masses for ATG8 swaps (ATG8-4-S1 and ATG8-4-S3) and PexRD54 expressed and purified in this study. (C) Table summarizing the thermodynamic and kinetic data that were extracted for each ITC run between the PexRD54 full-length, PexRD54 AIM peptide, and ATG8 swaps. (C) Replicates of ITC measuring the interaction between ATG8 swaps and the PexRD54 AIM peptide (left) and full-length protein (right). The top panels show heat differences upon injection of ligands and lower panels show integrated heats of injection (•) and the best fit (solid line) to a single site binding model using MicroCal PEAQ-ITC analysis software. ATG8, autophagy-related protein 8; ITC, isothermal titration calorimetry; SDS/PAGE, sodium dodecyl sulfate/polyacrylamide gel electrophoresis. (TIF) [file pbio.3000373.s011.tif]

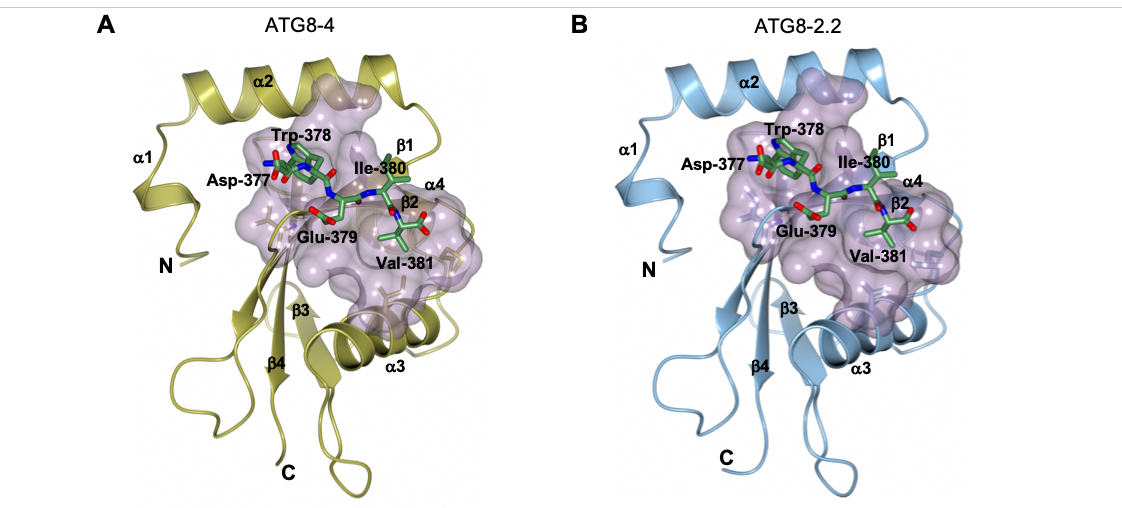

Supplement: S12 Fig — The molecular surface of each ATG8 that contacts the AIM peptide is shown in magenta. The AIM peptide is shown as a stick representation in each structure, with residues labelled. α-helices, β-strands, and N and C termini of ATG8-4 and ATG8-2.2 are labelled. AIM, ATG8-interacting motif; ATG8, autophagy-related protein 8. (TIF) [file pbio.3000373.s012.tif]

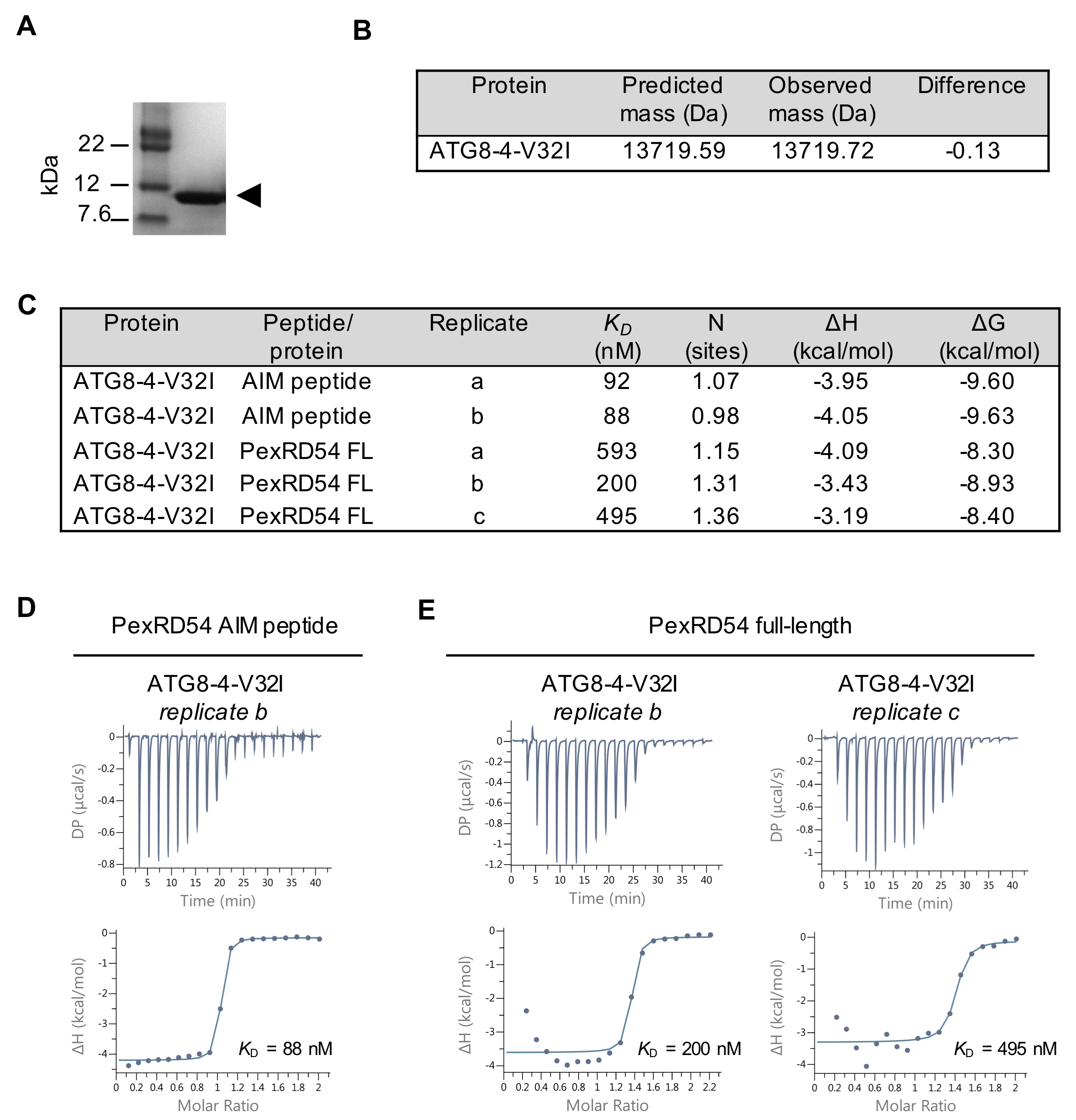

Supplement: S13 Fig — (A) Coomassie-stained SDS-PAGE showing purified ATG8-4-V32I. (B) Identity of ATG8-4-V32I was confirmed by measuring intact mass using MS. (C) Table summarizing the thermodynamic and kinetic data that were extracted for each ITC run between the PexRD54 full-length, PexRD54 AIM peptide, and ATG8-4-V32I. (D) Second replicate of the ITC trace showing interaction between ATG8-4-V32I and PexRD54 AIM peptide. (E) Replicates of the ITC traces showing interaction between ATG8-4-V32I and the full-length PexRD54. AIM, ATG8-interacting motif; ATG8, autophagy-related protein 8; ITC, isothermal titration calorimetry; MS, mass spectrometry; SDS-PAGE, sodium dodecyl sulfate/polyacrylamide gel electrophoresis. (TIF) [file pbio.3000373.s013.tif]

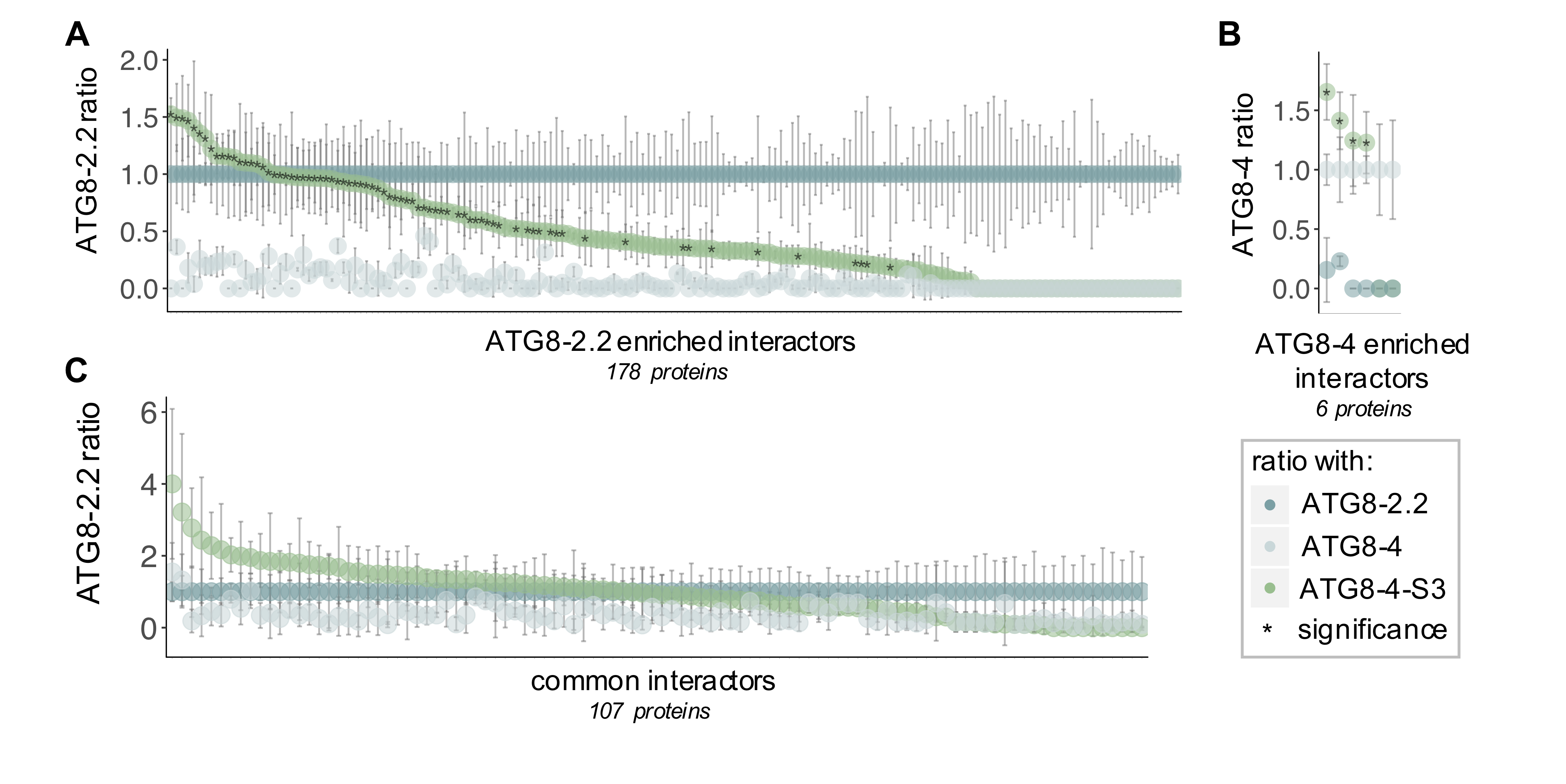

Supplement: S14 Fig — For each interactor in the dataset, the average peptide count data for ATG8-2.2 (teal), ATG8-4 (light gray), and ATG8-4-S3 (green) were normalized to either ATG8-2.2 or ATG8-4 data based on the enrichment category being analyzed: (A) values for ATG8-2.2–enriched interactors were normalized to ATG8-2.2, (B) values for ATG8-4–enriched interactors were normalized to ATG8-4, and (C) values for common interactors were normalized to ATG8-2.2. For (A) ATG8-2.2–enriched interactors and (B) ATG8-4–enriched interactors, this highlights the difference in how ATG8-2.2 and ATG8-4 interact with each protein in the set and how the ATG8-4-S3 interactions compare. For (A) ATG8-2.2–enriched interactors, the asterisk (*) marks proteins that showed no statistical difference in their interaction with ATG8-4-S3 as compared with ATG8-2.2 (in Fig 6d, “(+) S3 enrichment”); for (B) ATG8-4 enriched interactors, the asterisk (*) marks proteins that showed no statistical difference in their interaction with ATG8-4-S3 as compared with ATG8-4. For (C) common interactors, the graph highlights the similarity in how ATG8-2.2, ATG8-4, and ATG8-4-S3 interact with each protein in the set; due to the lack of statistical difference, this feature is not marked. ATG8, autophagy-related protein 8. (TIF) [file pbio.3000373.s014.tif]

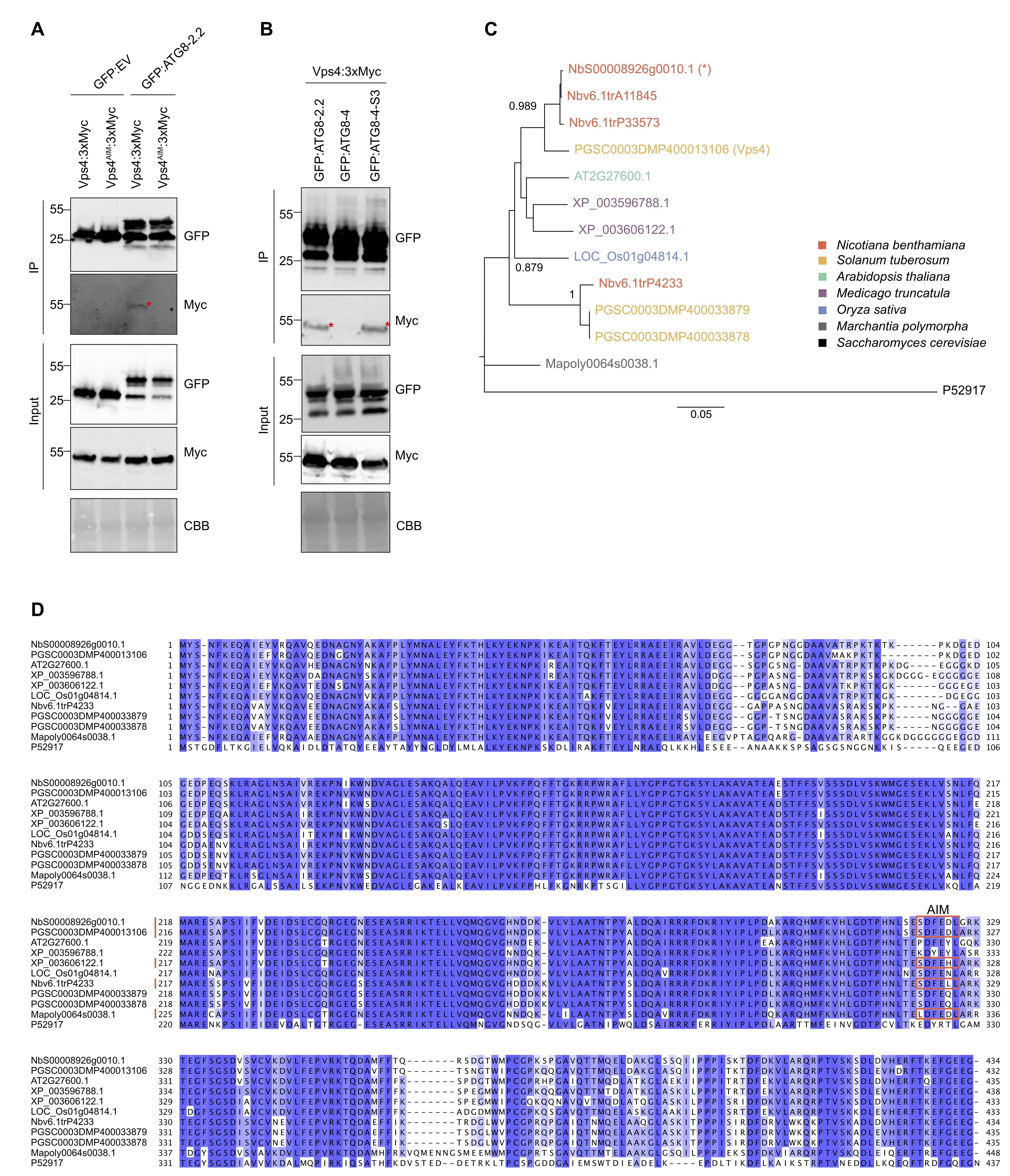

Supplement: S15 Fig — (A) Co-IP experiment between potato Vps4 and the Vps4 AIM mutant (Vps4AIM)—changing the AIM sequence of SDFEDL to SDAEDA—with ATG8-2.2. Vps4:3xMyc and Vps4AIM:3xMyc were transiently co-expressed with GFP:EV and GFP:ATG8-2.2, respectively. (b) Co-IP experiment between Vps4 and ATG8-2.2, ATG8-4, and ATG8-4-S3. Vps4:3xMyc was transiently co-expressed with GFP:ATG8-2.2, GFP:ATG8-4, and GFP:ATG8-4-S3. For (A-B), IPs were obtained with anti-GFP antiserum and total protein extracts were immunoblotted with appropriate antisera (listed on the right). Stars indicate expected band sizes. (C) Unrooted maximum-likelihood phylogenetic tree of orthologs of Saccharomyces cerevisiae (yeast) Vps4 from across select plant species, with the N. benthamiana Vps4 identified in the IP-MS experiment starred (*) and the S. tuberosum Vps4 tested in Co-IP experiments marked (“Vps4”). Colors indicate species, and bootstrap supports are noted when >0.7. The tree was calculated in MEGA7 [38] from a 448–amino acid alignment (MUSCLE [39], codon-based). The scale bar indicates the evolutionary distance based on amino acid substitution rate. (D) Alignment of Vps4 sequences included in the phylogenetic tree in (C), excluding the Nbv6.1trA11845 and Nbv6.1trP33573 sequences, which are almost sequence identical to NbS00008926g0010.1. The presence of a predicted AIM as determined by iLIR is marked with a red box [40]. AIM, ATG8-interacting motif; CoIP, co-immunoprecipitation; GFP:EV, green fluorescent protein empty vector; IP, immunoprecipitate; IP-MS, immunoprecipitation followed by mass spectrometry; Vps4, vacuolar protein sorting 4. (TIF) [file pbio.3000373.s015.tif]

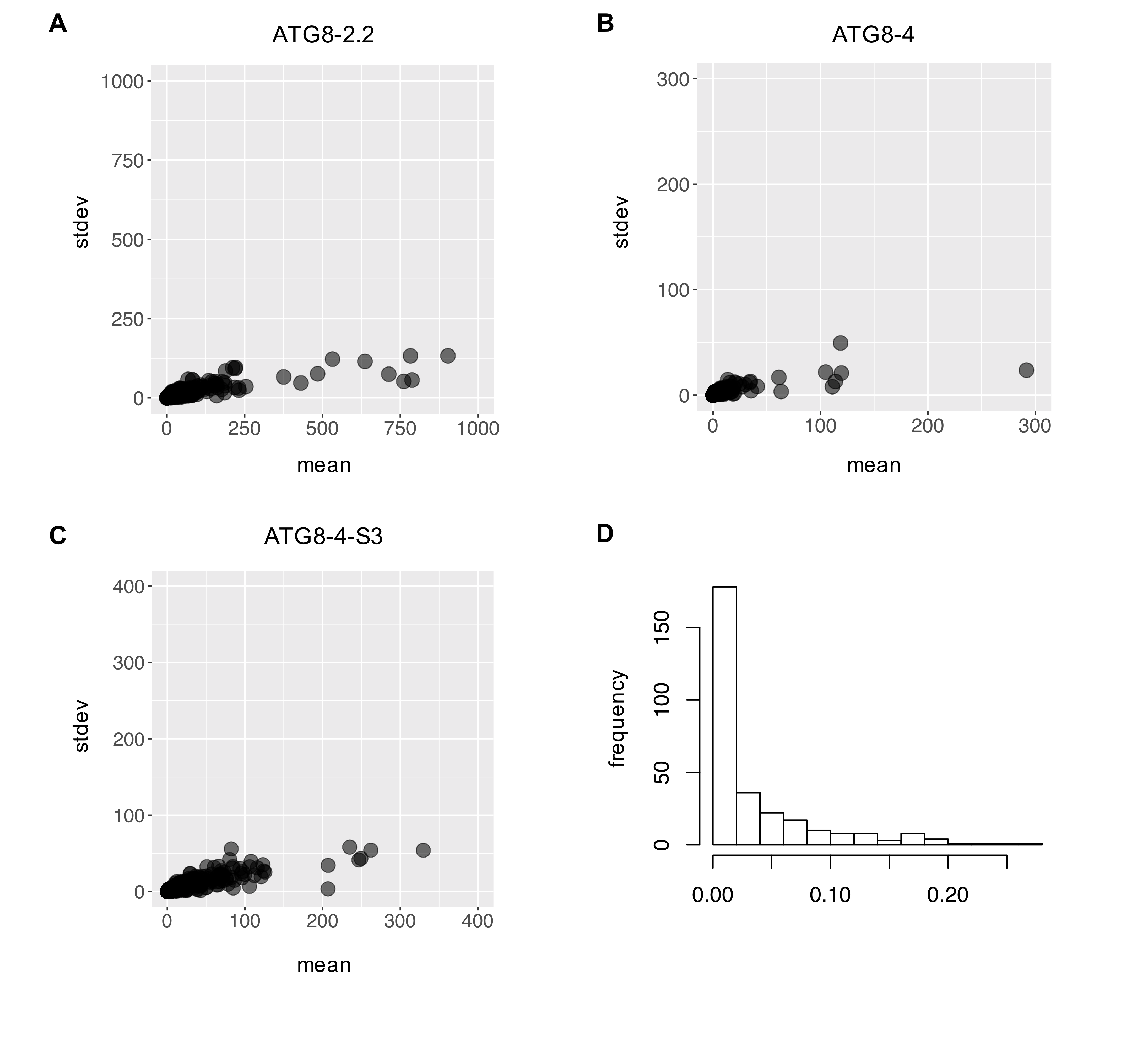

Supplement: S16 Fig — The standard deviation (stdev) versus mean is plotted for the GFP normalized peptide count data for three replicates of each construct tested in IP-MS, (A) ATG8-2.2, (B) ATG8-4, and (C) ATG8-4-S3, showing a normal distribution in each. (D) A histogram of ANOVA p-values showing the high level of significance within the dataset. ATG8, autophagy-related protein 8; GFP, green fluorescent protein; IP-MS, immunoprecipitation followed by mass spectrometry. (TIF) [file pbio.3000373.s016.tif]
